# Management affects the diversity and functions of root and leaf-associated microbiomes: implications for olive resilience

**DOI:** 10.3389/fpls.2025.1699667

**Published:** 2026-01-20

**Authors:** Emanuele Fosso, Gina Gizzi, Maria Tartaglia, Antonello Prigioniero, Maria Antonietta Ranauda, Maria Maisto, Mónica Labella-Ortega, Daniela Zuzolo, Carmine Guarino

**Affiliations:** Department of Science and Technology, University of Sannio, Benevento, Italy

**Keywords:** agricultural management, olive microbiome, olive root-associated microbiome, phyllosphere, leaf associated microbiome, rhizosphere

## Abstract

This study explores the impact of organic, conventional, and traditional agricultural management on the aboveground and belowground microbiomes of *Olea europaea* L. cv. Ortice, a cultivar widely cultivated in southern Italy’s agricultural landscape. Through metabarcoding analyses (16S rRNA and ITS), we assessed the influence of farming approaches on the microbiome traits of the olive holobiont. Our findings demonstrate that agricultural management practices significantly shape microbiome composition both aboveground and belowground. The conventional management was associated with the highest number of microbial biomarkers aboveground, mainly belonging to *Rhizobiaceae* and *Rhodocyclaceae* families. Instead, *Fusarium* (family *Nectriaceae*) was the most abundant taxon under organic treatment. Regarding root-associated microbiome, organic management supported a greater number of microbial biomarkers, including the bacterial genera *Actinophytocola* and *Streptomyces*, both known for their roles in promoting plant health and protecting against pathogens. In traditional systems, biomarkers included taxa from the order *Burkholderiales* and the species *Nocardioides islandensis*. Functional analysis of the aboveground fungal community revealed a higher capacity for endophytic interactions in traditional management, predominantly involving known pathogenic species such as *Alternaria alternata*, *Aureobasidium* spp., and *Cladosporium* spp. Similarly, traditional management was associated with significant enrichment of phototrophic functions belowground, mainly attributed to the bacterium *Rhodopseudomonas palustris*. Conversely, the potential for endophytic interactions was significantly greater under conventional management and was primarily linked to fungi within the class *Sordariomycetes*. Management practices shape distinct microbial communities both aboveground and belowground of olive groves, potentially influencing the resilience of Mediterranean agroecosystems and underscoring the importance of sustainable strategies.

## 1 Introduction

The olive tree (*Olea europaea* L.) has been a symbol of the Mediterranean for thousands of years, deeply impacting the region’s economy, society, and culture, while shaping its landscapes and identity (Brunori et al., 2020; Raz et al., 2024). Moreover, olive oil production is a vital economic sector in countries such as Italy, Spain, and Greece (Martins et al., 2024). Olive trees are well adapted to conditions of the Mediterranean climate, characterized by low rainfall and rocky, nutrient-poor soils, and they have evolved to thrive in these environments (Erel et al., 2013). Nevertheless, the health and productivity of olive trees are profoundly influenced by the management practices employed in olive groves, which can have significant effects on soil quality as well as plant growth (Orlandi et al., 2020). In this context, the type of management has changed profoundly over time to meet the growing product demand and to address climate challenges and olive farming has undergone significant intensification, marked by the adoption of irrigation systems, artificial fertilizers, and pesticides (Infante-Amate et al., 2016).

Olive groves are typically managed under three main systems: organic, traditional, and conventional farming (Cárdenas et al., 2015). The organic system follows strict guidelines established by legislation for biological agriculture, which prohibit the use of synthetic chemicals (EC, 2007). Organic farming practices focus on promoting biodiversity, enhancing soil fertility through crop rotation, composting, and natural pest control methods (the fungicides are mineral or natural as approved under EU regulation), and supporting a sustainable farming model (Mäder et al., 2002). In contrast, traditional olive farming represents a low-intervention system in which management practices are minimal and mostly limited to essential activities such as pruning and basic soil maintenance. Farmers typically avoid the use of synthetic fertilizers, or other chemical inputs, relying instead on natural ecosystem processes to sustain plant health. This approach seeks to maintain the ecological balance of the orchard, promoting long-term resilience of the trees and preserving the native microbial communities associated with both soil and plant tissues (Fekete et al., 2023). Unlike organic and traditional management, in conventional olive farming, the use of synthetic chemicals, including fertilizers, pesticides, and herbicides, to maximize yields and manage pests is adopted. In conventional management foliar spray of synthetic fungicides are often used to control olive leaf spot and olive scab in early spring to protect the new emerging leaves, providing both preventive and early curative activity. In addition, soil tillage is carried out more frequently and more deeply than in traditional and organic treatments. This approach is designed to maximize productivity, but it may have adverse effects on the soil ecosystem and plant associated microbiome (Garbeva et al., 2008). Organic farming is typically associated with a greater diversity of soil microorganisms and a higher prevalence of beneficial microbes. For example, it has been observed that this system often foster the presence of nitrogen-fixing bacteria, mycorrhizal fungi, and microorganisms involved in nutrient cycling and disease suppression (Mäder et al., 2002; Iqbal et al., 2023). Conventional farming, by contrast, with its reliance on chemical inputs and more intensive soil management practices, can lead to a shift of microbial communities, which may have negative consequences for soil fertility and plant health. Traditional farming systems, which involve limited interventions (such as pruning, the soil is left untilled, with periodically cut of natural herbaceous cover) without additional chemicals or fertilizers, can strike a balance between maintaining soil quality and ensuring the sustainability of the farming system (Khmelevtsova et al., 2022). A growing body of research has highlighted the importance of soil management in determining the structure and function of microbial communities. For instance, Wentzien et al. (2023) demonstrated that different soil management practices, such as tillage and cover cropping, had significant effects on microbial diversity and functionality in olive groves. They found that organic farming systems supported a more diverse microbial community, contributing to improved soil quality and plant health. Similarly, Montes-Borrego et al. (2013) showed that organic farming practices in olive groves promoted higher microbial diversity, especially among bacterial groups involved in nutrient cycling and disease suppression. These studies illustrate the importance of agricultural practices in shaping both microbial diversity and functionality within the agricultural ecosystem and playing a critical role in maintaining soil health and plant productivity. The olive plant hosts a complex and diverse microbiome in both its aerial and belowground compartments. In line with the broader concept of root and leaf associated microbiome, these communities, primarily bacteria and fungi, can colonize plant tissues epiphytically, on external surfaces, or endophytically, within internal tissues, forming an integrated network of plant-microbe interactions across the entire plant (Larry et al., 2016; Sinha et al., 2025; Zhu et al., 2022). Microorganisms engage in intricate interactions with each other and with their host, influencing key plant traits, including growth, nutrient uptake, and disease presence or resistance (Berendsen et al., 2012). The phyllosphere harbors a wide range of microbial taxa, including both pathogenic and beneficial microorganisms. While some members of the phyllosphere microbiota can act as pathogens and compromise plant health, others contribute positively to the host by suppressing disease. Beneficial phyllosphere microbes can limit pathogen infection through direct mechanisms, such as the secretion of antagonistic metabolites and competition for space and nutrients, or indirectly, by priming or inducing plant immune responses (Fontana et al., 2021; Morales-Cedeño et al., 2021; Binyamin et al., 2019). Similarly, root-associated microbiome can play a pivotal role in shaping plant health by modulating nutrient cycling, enhancing stress tolerance, and mediating pathogen suppression (Berendsen et al., 2012). The majority of studies on the olive tree microbiome have investigated its composition in individual plant compartments (Fernández-González et al., 2019; Malacrinò et al., 2022). While some authors have explored the connections between aboveground and belowground microbiomes (e.g., Kakagianni et al., 2023; Martins et al., 2024), more research to understand how agricultural management practices may shape such interactions is needed. Given the crucial role of the plant microbiome in determining host fitness and function, there is increasing interest in characterizing, modeling, and managing host-microbiome interactions to optimize plant performance and resilience. This has led to the emergence of microbiome-informed strategies in agriculture, where microbiome-driven cropping systems are being developed as a means to enhance crop productivity and sustainability. Such approaches hold the potential to drive a paradigm shift in plant production, contributing to a more resilient and eco-friendly agricultural future.

With this study, we aimed to add new knowledge about whether adaptation to contrasting management systems alters the microbiome community composition and functionality of above- and below-ground olive trees, focusing on the cultivar Ortice which is highly representative of a key Mediterranean region for oil production such as Southern Italy. To achieve this target, the following objectives were pursued: assessment of the impact of management (traditional, organic and conventional) in (i) shaping the belowground root-associated (including the endorhizosphere, rhizoplane, and ectorhizosphere) microbial (bacteria and fungi) community, (ii) shaping the aboveground leaf-associated (including both the phylloplane and internal endophytic microbial (bacteria and fungi) community and (iii) altering the potential functional traits of the root and leaf associated bacterial and fungal community. We test the hypothesis that low-impacting management has a positive influence on the belowground and/or aboveground microbial communities associated with olive trees at different levels, such as diversity and functional profile.

## 2 Materials and methods

### 2.1 Sampling activity

**Figure 1:**
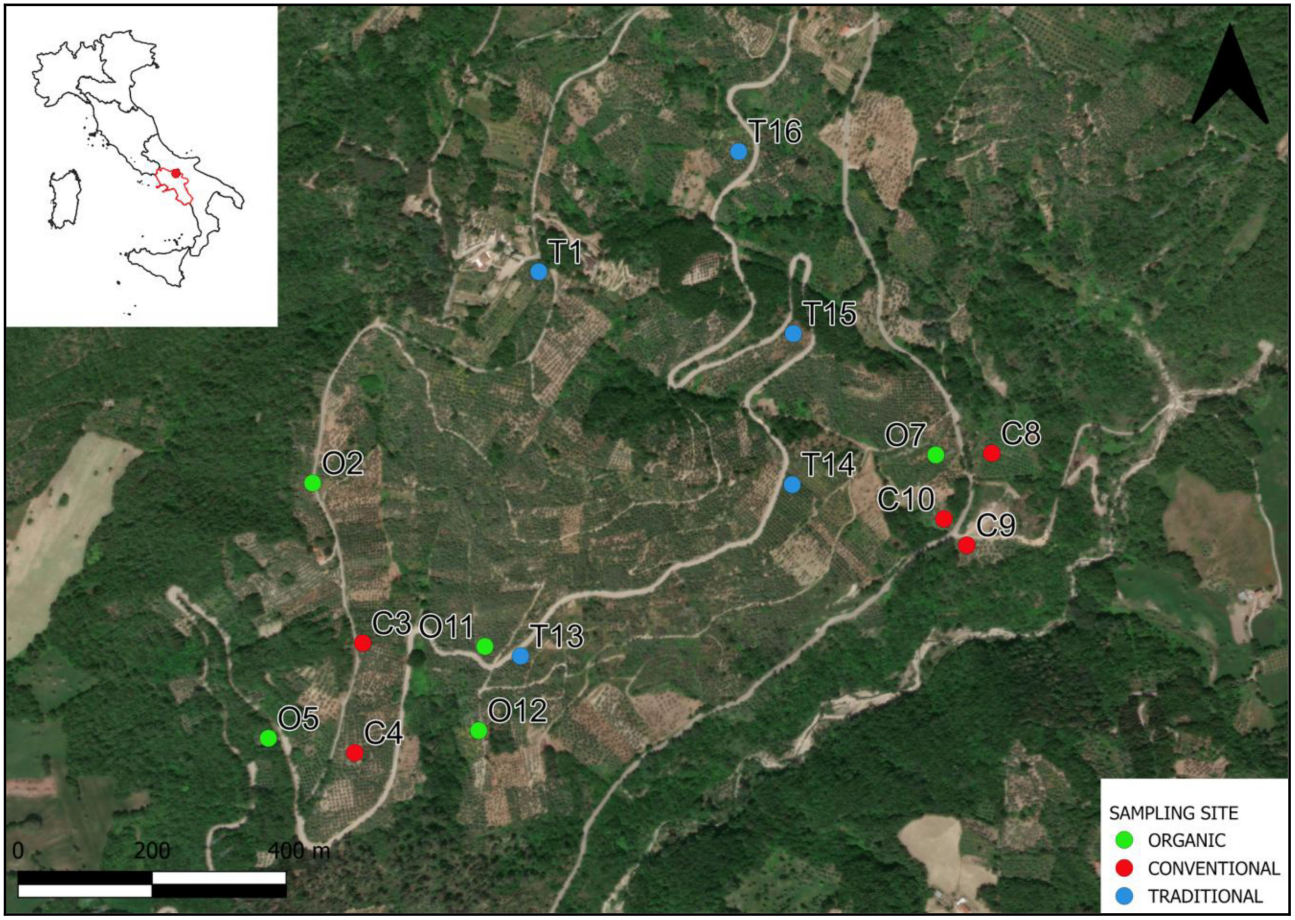
Study area and sampling points. O, fields with organic treatment, C, fields with conventional treatment, T, fields with traditional treatment.

All samples analyzed in the study were collected from 15 olive groves of *Olea europaea* L. cv Ortice in March 2024. The study area falls within the Sannio olive cultivation area (Southern Italy, **Figure 1**). All fields are located in areas with similar climatic conditions and soil types (clayey and marly hill soil type, Campania Region Geoportal), at 600 m above sea level. The physical-chemical features of the soil are reported in **Supplementary Table S1** (**Supplementary Material**). Five olive groves were selected for each type of the considered agricultural management: organic, traditional, and conventional. During the sampling period, organic groves were in the final stage of winter cover-crop growth (which consisted mainly of legume-based green manure mixtures) with no recent soil disturbance. In conventional groves, late-winter tillage and weed-control operations had recently been carried out, and preventive fungicide applications targeting newly emerging leaves occurred. In addition, nitrogen-rich fertilizers in late winter/early spring were applied to stimulate vegetative growth. Traditional farming systems had no recent mechanical inputs (e.g., cutting of the natural herbaceous cover) prior to sampling. The olive groves across the three farming systems were similar in size, each covering roughly 2,000 m². The average distance between sampled groves was 590 m, and together the 15 groves spanned a total geographic area of 0.48 km². At each olive grove, roots (including the endorhizosphere, rhizoplane, and ectorhizosphere) and leaves (including both the phylloplane and endophytic) samples were collected in order to perform metagenomic analyses, respectively. To ensure the representativeness of the olive grove and to consider biological variability, 6 trees of the same age were identified for each grove running along the diagonal of the grove in the NE - SO direction avoiding border interferences. To collect the belowground samples, the topsoil layer (less than 5 cm) was removed, and then the main roots of each plant were tracked until young and cork-free roots were discovered at a depth of approximately 5-40 cm. Using a core drill (or soil auger), six sub-samples consisting of roots with attached soil were collected at each site. Fully expanded leaves were sampled from the same individuals, collecting from branches with similar light exposition and position in the canopy. During sampling, all equipment was sterilized (10% bleach followed by 70% Ethanol and then distilled water) to avoid cross-contamination between sample types and between fields. The six sub-samples (per type) were homogenized to obtain a composite sample for each grove.

After collecting, all samples were transported at 4°C to the Department of Science and Technology at the University of Sannio and then stored at -80°C until analysis.

### 2.2 DNA extraction

Microbial communities root and leaf associated were analyzed through total DNA extraction from composite plant samples. For the root-associated communities, approximately 0.2 g of roots with tightly adhering soil were collected. Root systems were gently shaken to remove loosely attached soil particles, retaining only the soil fraction in close contact with the root surface. The resulting material, consisting of root tissues and the adherent soil fraction, was homogenized under sterile conditions to ensure an even representation of microorganisms inhabiting both endorhizosphere, rhizoplane, and ectorhizosphere. This sampling strategy was designed to capture the overall microbial assemblage directly interacting with the root environment. For the leaf-associated microbiome analyses, DNA was extracted from entire leaf tissues rather than from surface washes or detached cells. This approach was adopted to include both epiphytic (surface-dwelling) and endophytic (internal) microorganisms, thereby representing the complete microbial assemblage associated with the leaf environment. Leaf tissues were processed under sterile conditions and homogenized prior to extraction to obtain a composite sample encompassing both microbial fractions.

Total genomic DNA from all samples was isolated using the E.Z.N.A.^®^ Soil DNA Kit (Omega Bio-tek, Inc.) following the manufacturer’s instructions. DNA concentration was quantified fluorometrically with a Qubit system (Thermo Fisher Scientific), and DNA integrity was assessed using the 4150 TapeStation system (Agilent Technologies). The purified DNA samples were subsequently used for downstream analyses of microbial community composition and predicted functional potential.

### 2.3 Metagenomic analysis

The bacterial community was analyzed by amplifying the 16S rRNA genes by PCR using primers for the hypervariable regions V2, V3, V4, V6-7, V8 and V9, according to the Ion 16S Metagenomics kit (Life Technologies, 5781 Van Allen Way Carlsbad, CA, USA 92008). The kit used to amplify the resulting libraries was the Ion Plus Fragment Library kit (Thermo Fisher Scientific) while with the Qubit 3.0 fluorometer (Thermo Fisher Scientific, Carlsbad, CA) using the Qubit DNA High Sensitivity Assay kit (Thermo Fisher Scientific), the libraries were quantified. The libraries were pooled with a final equimolar concentration of 40 pM and they were loaded onto chips for sequencing with the Ion Torrent™ system, Ion S5™, using the Ion 530™ Chip kit. Ion GeneStudio™ S5 was subsequently used to sequence the templates. The Ion Reporter™ Software 5.20 platform with the Metagenomics 16S w1.1 v5.14 workflow was used to analyze the generated unaligned binary data files (Binary Alignment Map, BAM) (Ranauda et al., 2024). The Ion reporter workflow is based on both the MicroSEQ™ ID 16S Reference Library v2013.1 and Greengenes v13.5 as reference databases. Any reads shorter than 150 base pairs were excluded after primer trimming. The analysis was performed with a Minimum Alignment Coverage of 90% and a Read Abundance Filter set to 10, with a 99% identity threshold. Finally, the 16S rRNA sequence reads were assigned to operational taxonomic units (OTUs) through the closed reference method. For the analysis of the fungal community, the ITS1 and ITS2 regions were amplified using multiplex PCR. The specific primer sequences used are as follows: ITS1 Fw: GTCCCTGCCCTTTGTACACA; ITS1 Rev: TTTCGCTGCGTTCTTCATCG and ITS2 Fw: GTGAATCATCGAATCTTTGAA; ITS2 Rev: TTCCTCCGCTTATTGATATGC. The amplification reactions were carried out with the TaqMan™ Environmental Master Mix 2.0 (Applied Biosystems™). The PCR protocol was as follows: 3 minutes at 94°C, followed by 25 cycles of 15 seconds at 94°C, 30 seconds at 50°C, and 60 seconds at 68°C, with a final extension at 72°C for 7 minutes. The generated libraries were amplified and sequenced using the same procedure as the 16S libraries. Taxonomic identification was performed using the UNITE database, applying a length filter of >10 bp. Any reads shorter than 150 base pairs were excluded after primer trimming. The analysis was conducted with a Minimum Alignment Coverage of 90% and a Read Abundance Filter set to 10, with a 99% identity threshold. The ITS sequence reads were then grouped into operational taxonomic units (OTUs) using the closed-reference method. All sequencing data have been deposited in the NCBI with accession number SUB15436629.

### 2.4 Data analysis

The microbial community was analyzed using R version 4.3.3 (R Development Core Team, 2024), with several key packages including *reshape2* (v.1.4.4), *ggplot2* (v.3.4.2), *plyr* (v.1.8.8) (Wickham, 2007; Wickham and Sievert, 2009, 2011), *Hmisc* (v.5.1–0) (Harrel and Dupont, 2019), and *phyloseq* (v.1.42.0) (McMurdie and Holmes, 2013). Both 16S and ITS sequences were processed into OTUs and imported into R as *phyloseq* objects for exploratory analysis and to evaluate the taxonomic composition of the samples. The *phyloseq* object includes the taxonomy table (indicating the taxonomic classification of each OTU), the OTU table (which records OTU counts per sample) and the sample data matrix (providing metadata for each sample). To homogenise the number of sample reads (read depth) and avoid bias in distance and dissimilarity calculations (Gloor et al., 2017), we used the function microbiome::transform from the *microbiome* package in R with the method “compositional”; then the data was prepared following the procedure described by Zuzolo et al. (2023). To assess the dispersion of microbial communities within management systems, the *betadisper* function (*vegan* package) was used since it computes the distance of each sample to the centroid of its group, providing a measure of within-treatment variability. Statistical significance of differences in dispersion among treatments was tested using permutation tests (Permutation test for homogeneity of multivariate dispersions with 999 permutations). Mean distance-to-centroid values by treatment were extracted from the *betadisper* results to summarize the within-group variability (Maher et al., 2019). Shannon diversity index values were calculated for all microbial communities in both compartments. For each management system, mean values and standard deviations from five samples were computed and differences in alpha diversity among treatments were assessed using one-way ANOVA. To evaluate dissimilarity in microbial communities between treatments, we performed first the Permutational analysis of variance (PERMANOVA) with *adonis* function from *vegan* package and then the *post-hoc* test *pairwise.adonis* function from *pairwiseAdonis* package. The R/Bioconductor package microbiomeMarker (Cao et al., 2022) was employed to investigate taxa that exhibit differential abundance and to identify microbial biomarkers characterizing microbiome data of the different management type. In particular, linear discriminant analysis effect size (LEfSe) was utilized for the discovery of metagenomic biomarkers after data normalization using the total sum scaling (TSS) method. The parameters employed for the LEfSe analysis were as follows: Kruskal–Wallis test with α = 0.05 to identify features significantly different among management systems. LDA score cutoff of ≥ 3.0 (log10 scale) to define biomarkers. Multiple testing correction: the default LEfSe workflow was used; no additional FDR adjustment was applied. Additionally, functional predictions were made using the R package microeco (Liu et al., 2021). The bacterial functional profile was predicted using FAPROTAX, a tool designed for the functional annotation of prokaryotic taxa. Following this, fungal OTUs were annotated with functional data by cross-referencing them with the FungalTraits database (Põlme et al., 2020). To assess differences in functional traits across various sample types, an ANOVA test (p < 0.05) was performed. All results were visualized using the ggplot2 package. Both FAPROTAX (Louca et al., 2016) and FungalTraits (Põlme et al., 2020) are comprehensive databases containing taxonomic and functional descriptions derived from a selection of carefully curated publications (Wen et al., 2023).

## 3 Results

### 3.1 Sequencing results

For 16S metagenomic analysis across 15 samples (5 samples per management type) of leaf-associated microbiome, a total of 8,817,677 reads were generated, of which 7,237,936 were valid reads (ranging from a minimum of 248,470 to a maximum of 720,015 per sample). Additionally, 5,516,331 reads were successfully mapped. For the ITS metagenomic analysis, a total of 5,756,513 reads were obtained from 15 samples (ranging from 68,054 to 641,050 reads per sample), considering a blast abundance threshold >10. Regarding the sequencing data of the root-associated microbial community the 16S metagenomic analysis yielded a total of 7,030,958 reads, of which 5,882,827 were valid, across 15 samples (5 per management type), with 2,153,701 reads successfully mapped. In contrast, the ITS analysis detected a total of 7,703,087 reads distributed across the 15 samples. The sequencing depth (**Supplementary Figure S1**, **Supplementary Material**) was sufficient for both microbial communities, as all rarefaction curves approached a clear plateau, indicating adequate coverage of bacterial and fungi richness.

### 3.2 Composition of leaf-associated bacterial and fungal communities

From the taxonomic assignment with the NCBI database, we found that the data of the aboveground compartment overall returns 98 bacterial OTUs, divided into 6 phyla, 10 classes, 27 orders, 47 families, 67 genera and 86 species. The fungi contained 151 OTUs, divided into 3 phyla, 15 classes, 34 orders, 63 families, 84 genera and 80 species (**Supplementary Table S2**). In terms of relative abundance, *Pseudomonadota, Actinomycetota* and *Bacteroidota* are the most abundant bacterial phyla in all treatments (respectively: 0.49 ± 0.07, 0.27 ± 0.06, 0.22 ± 0.11 in the organic, 0.43 ± 0.04, 0.25 ± 0.04, 0.27 ± 0.05 in the conventional and 0.44 ± 0.11, 0.48 ± 0.11, 0.07 ± 0.09 in the traditional) (**Supplementary Figure S3a**). *Streptomycetaceae, Fulvivirgaceae* and *Chitinophagaceae* are the most abundant families in the organic (respectively: 0.14 ± 0.08, 0.11 ± 0.06, 0.07 ± 0.03) and conventional treatments (respectively: 0.15 ± 0.06, 0.14 ± 0.02, 0.10 ± 0.04); the traditional treatment differs with *Micrococcaceae, Streptomycetaceae* and *Sphingomonadaceae* being the most abundant (respectively: 0.35 ± 0.15, 0.22 ± 0.14, 0.21 ± 0.12) (**Supplementary Figure S3b**). Regarding the aboveground bacterial species, *Arthrobacter russicus*, *Sphingomonas* sp. and *Ralstonia insidiosa* are the most abundant of traditional treatments (respectively: 0.21 ± 0.15, 0.13 ± 0.12, 0.09 ± 0.11). Instead, *Ohtaekwangia kribbensis* is the most abundant species in organic (0.11 ± 0.06) and conventional treatments (0.14 ± 0.02); the second most abundant species are *Actinacidiphila glaucinigra* for organic (0.08 ± 0.05) treatment and *Niastella yeongjuensis* for conventional treatment (0.08 ± 0.03) (**Supplementary Figure S3c**). *Ascomycota* mainly dominated the leaf-associated fungal community across all agricultural treatments, showing almost complete prevalence in organic (1.00 ± 0.00), conventional (0.98 ± 0.02) and traditional samples (0.98 ± 0.01). In contrast, *Basidiomycota* were present only at very low relative abundances in all treatments (≤ 0.02 ± 0.01) (**Supplementary Figure S4a**). The *Phaeosphaeriaceae* family is the most abundant in organic (0.62 ± 0.25) and conventional (0.48 ± 0.33) treatments, while in the traditional is *Pleosporaceae* (0.59 ± 0.36), however, *Phaeosphaeriaceae* (0.34 ± 0.28) are also well represented in these samples (**Supplementary Figure S4b**). *Parastagonospora avenae* species is the most abundant in organic (0.63 ± 0.24) and conventional (0.46 ± 0.36) treatments, on the other hand, *Stemphylium solani* is the dominant species in traditional treatment samples (0.58 ± 0.36) (**Supplementary Figure S4c**). Aboveground bacterial α-diversity (**Table 1**) differed significantly among management systems, with the traditional system showing reduced diversity, while fungal α-diversity did not vary. The dispersion of microbial communities within treatments, evaluated using the mean distances to centroid reported in **Supplementary Table S3** of the **Supplementary Material**, showed no significant differences within management systems. Both the centroid distances and the associated permutation tests indicated no significant variation in dispersion (betadisper, all p > 0.05). Instead, the pairwise PERMANOVA results (**Table 2**, **Supplementary Figure S5**) indicate significant differences of the leaf-associated bacterial community. Specifically, the traditional treatment shows significant differences compared to both the organic treatment (p = 0.015) and the conventional treatment (p = 0.013). For fungi, traditional management significantly differs from the organic treatment (p = 0.045), but the difference with the conventional treatment is marginal (p = 0.093).

**Table 1:** Mean, standard deviation (sd) and p-values of one-way ANOVA of Shannon index for bacterial and fungal communities within management (Conventional, Organic, Traditional).

		Treatment	Shannon (mean ± sd)
Leaf-associated	Bacteria	Conventional	3.12 ± 0.18
		Organic	3.11 ± 0.16
		Traditional	2.01 ± 0.59
		*p value*	5.33x10^-0.4^
	Fungi	Conventional	1.63 ± 0.5
		Organic	1.64 ± 0.17
		Traditional	1.18 ± 0.4
		*p value*	0.11
Root-associated	Bacteria	Conventional	3.30 ± 1.47
		Organic	4.06 ± 1.27
		Traditional	3.59 ± 0.98
		*p value*	0.64
	Fungi	Conventional	2.54 ± 0.47
		Organic	3.43 ± 0.08
		Traditional	3.31 ± 0.63
		*p value*	0.01

**Table 2:** Beta diversity results.

	Leaf-associated					
	Bacteria			Fungi		
Indicators	T vs O	T vs C	O vs C	T vs O	T vs C	O vs C
Bray-Curtis	0.018	0.009	0.848	0.037	0.049	0.866
Jaccard	0.015	0.013	0.826	0.045	0.093	0.865
	Root-associated					
	Bacteria			Fungi		
Indicators	T vs O	T vs C	O vs C	T vs O	T vs C	O vs C
Bray-Curtis	0.204	0.359	0.169	0.373	0.044	0.068
Jaccard	0.169	0.302	0.167	0.383	0.045	0.043

The *p value* of pairwise comparisons for bacterial and fungal communities within management Conventional (C), Organic (O) and Traditional (T) for Bray-Curtis and Jaccard distances are shown.

### 3.3 Composition of root-associated bacterial and fungal communities

Among the 15 samples from the root-associated microbiome, we identified 785 bacterial OTUs belonging to 14 phyla, 25 classes, 62 orders, 125 families, 229 genera and 674 species. Fungal OTUs were 608 divided into 8 phyla, 33 classes, 91 orders, 184 families, 304 genera and 301 species (**Supplementary Table S4**).

When considering relative abundance, the root-associated bacterial community shows a clear dominance of the phyla *Pseudomonadota* and *Actinomycetota* across all treatments, with *Pseudomonadota* accounting for 0.57 ± 0.04 in the organic, 0.53 ± 0.28 in the conventional, and 0.63 ± 0.11 in the traditional treatment, and *Actinomycetota* representing 0.39 ± 0.04, 0.23 ± 0.17, and 0.32 ± 0.10 in the same treatments, respectively (**Supplementary Figure S6a**). The *Sphingomonadaceae* family is the most abundant in traditional (0.23 ± 0.09) and organic (0.21 ± 0.08) treatments, while in conventional the *Bacillaceae* family is the most abundant (0.19 ± 0.41)(**Supplementary Figure S6b**). Similarly, the genus *Sphingomonas* is the most abundant in all samples (respectively: 0.19 ± 0.07 in the traditional, 0.18 ± 0.09 in the organic and with 0.13 ± 0.08 treatment). In terms of species, the most abundant in traditional (0.08 ± 0.04) and organic (0.05 ± 0.02) treatments is *Enhydrobacter aerosaccus*, while *Caldibacillus thermoamylovorans* presents a relative abundance of 56% in the C8 sample of the traditional treatment. (**Supplementary Figure S6c**). The root-associated fungal community in all samples across the three management practices is predominantly composed of three phyla: *Ascomycota* (with a relative abundance of: 0.65 ± 0.08 in organic treatment, 0.82 ± 0.17 in conventional and 0.75 ± 0.06 in traditional), *Basidiomycota* (with a relative abundance of: 0.23 ± 0.09 in organic treatment; 0.14 ± 0.16 in conventional and in 0.75 ± 0.15 traditional), and *Mucoromycota* (with a relative abundance of: 0.11 ± 0.02 in organic treatment; 0.04 ± 0.02 in conventional and in 0.48 ± 0.10 traditional) (**Supplementary Figure S7a**). The family with the highest relative abundance is *Nectriaceae* (phylum *Ascomycota*), with a relative abundance of 0.14 ± 0.09 in the traditional treatment, 0.19 ± 0.08 in the organic treatment, and 0.39 ± 0.29 in the conventional treatment (**Supplementary Figure S7b**). In fact, *Fusarium proliferatum* is the most abundant species in the conventionally treated fields (with 0.29 ± 0.24) (**Supplementary Figure S6c**). Belowground, bacterial α-diversity was similar across treatments, whereas fungal α-diversity was higher under organic management (**Table 1**). Both the centroid distances and the associated permutation tests indicated no significant variation in dispersion (*betadisper*, all p > 0.05) within treatment (**Supplementary Table S3**). The pairwise PERMANOVA (**Table 2**, **Supplementary Figure S5**) shows no significant differences in the composition of the root-associated bacterial community among the three treatments (p > 0.1). However, the fungal community in the conventional treatment differs significantly when compared to both the traditional (p = 0.045) and organic (p = 0.043) treatments.

### 3.4 Leaf-associated biomarkers of agricultural management types

**Figure 2:**
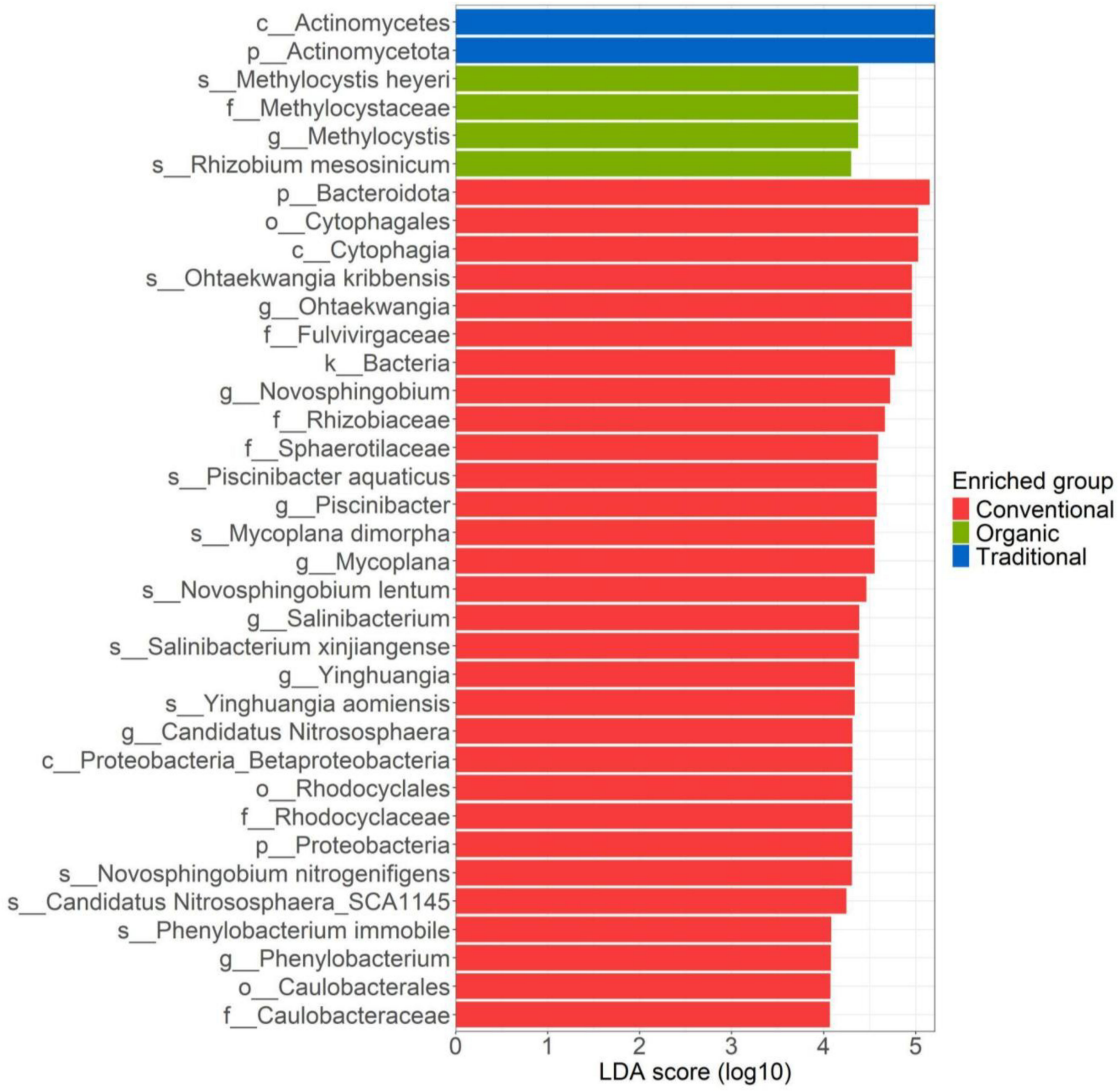
LefSe analysis of the leaf-associated bacterial community for the three managements: conventional, traditional and organic.

LefSe analysis identified a total of 35 microbial biomarkers across the three management regimes (**Figure 2**). Among these, the earliest differentiating taxa were associated with traditional management (blue), with the class *Actinomycetes* and its parent phylum *Actinomycetota* (LDA scores > 5). In contrast, conventional management accounted for the majority of differentially abundant taxa, comprising 28 distinct biomarkers. The five most representative biomarkers under conventional management belonging to the phylum *Bacteroidota*, and included the class *Cytophagia*, order *Cytophagales*, genus *Ohtaekwangia*, species *Ohtaekwangia kribbensis*, and the family *Fulvivirgaceae*, all exhibiting LDA scores around 5. While not individually emphasized by the analysis, a considerable proportion of biomarkers associated with conventional management were taxonomically affiliated with the phylum *Pseudomonadota*, suggesting its potential relevance in shaping aboveground bacterial communities under this regime. Indeed, ranking the taxa according to their own scores, we find: the genus *Novosphingobium* and the families *Rhizobiaceae* and *Sphaerotilaceae* (LDA scores of 4.7 and 4.6, respectively); the genus *Piscinibacter* and the species *Piscinibacter aquaticus* (LDA scores of about 4.6); the genus *Mycoplana* and the species *Mycoplana dimorpha* (LDA scores of about 4.5) and the species *Novosphingobium lentum* with an LDA of 4.5. The genus *Salinibacterium*, including the species *Salinibacterium xinjiangense*, and the genus *Yinghuangia* with the species *Yinghuangia aomiensis*, all belonging to the phylum *Actinomycetota*. Within conventional management, we also find the class *Proteobacteria*, the order *Rhodocyclales*, and the related family *Rhodocyclaceae*. Additionally, two taxa from the kingdom *Archaea*, *Candidatus Nitrososphaera* and the species *Candidatus Nitrososphaera SCA1145*, with LDA scores of approximately 4.4 and 4.3, respectively, are also highlighted. The final biomarkers identified through LefSe analysis associated with conventional management include the species *Phenylobacterium immobile*, the genus *Phenylobacterium*, and the family *Caulobacteraceae*, all with LDA scores near 4. Lastly, the biomarkers shown in green in **Figure 2** correspond to the third type of management: organic management. These include the genus *Methylocystis*, the family *Methylocistaceae*, as well as the species *Methylocystis heyeri* and *Rhizobium mesosinicum*, all with LDA scores around 4.3 (**Figure 2**).

**Figure 3:**
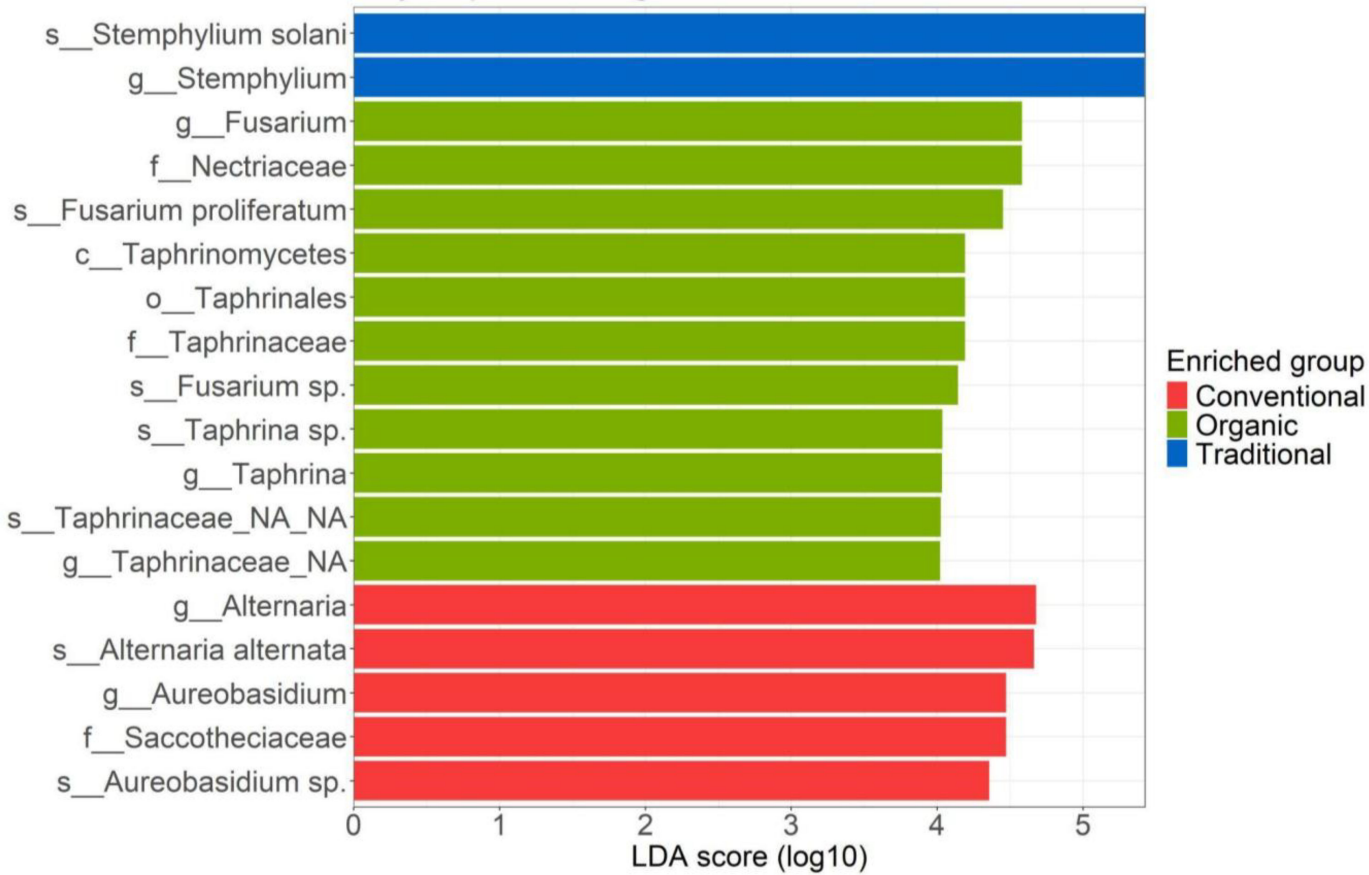
LefSe analysis of the leaf-associated fungal community for the three managements: conventional, traditional and organic.

Analyzing the aboveground fungal community, the LefSe analysis identified 18 differentially abundant biomarkers among the three soil treatment types (**Figure 3**). Regarding the fungal biomarkers of organic management, the most relevant, is the family *Nectriaceae* (*Ascomycota* phylum), with an LDA score exceeding 4.5. Within this family, the genus *Fusarium*, and the species *Fusarium proliferatum* and *Fusarium* sp.(with LDA scores slightly over 4) are identified as biomarkers. Additional biomarkers highlighted by LefSe include the class *Taphrinomycetes*, the order *Taphrinales*, the family *Taphrinaceae*, the genus *Taphrina*, and the species *Taphrina* sp., all exhibiting LDA scores just above 4. In the conventional management shown in red the most representative differentially abundant biomarkers are members of the orders *Pleosporales* and *Dothideales*. In the order *Pleosporales*, the differentially most abundant taxa are represented by the genus *Alternaria* and the species *Alternaria alternata* with a score of around 4.5. For the order *Dothideales*, on the other hand, the family *Saccotheciaceae*, the genus *Aureobasidium* and the species *Aureobasidium* sp. Are the representative biomarkers. Finally, regarding the traditional management (shown in blue), only two taxa resulting from the Lefse analysis belong exclusively to the phylum *Ascomycota*; the genus *Stemphylium* and the species *Stemphylium solani* with an LDA of 5 (**Figure 3**).

### 3.5 Root-associated biomarkers of agricultural management types

**Figure 4:**
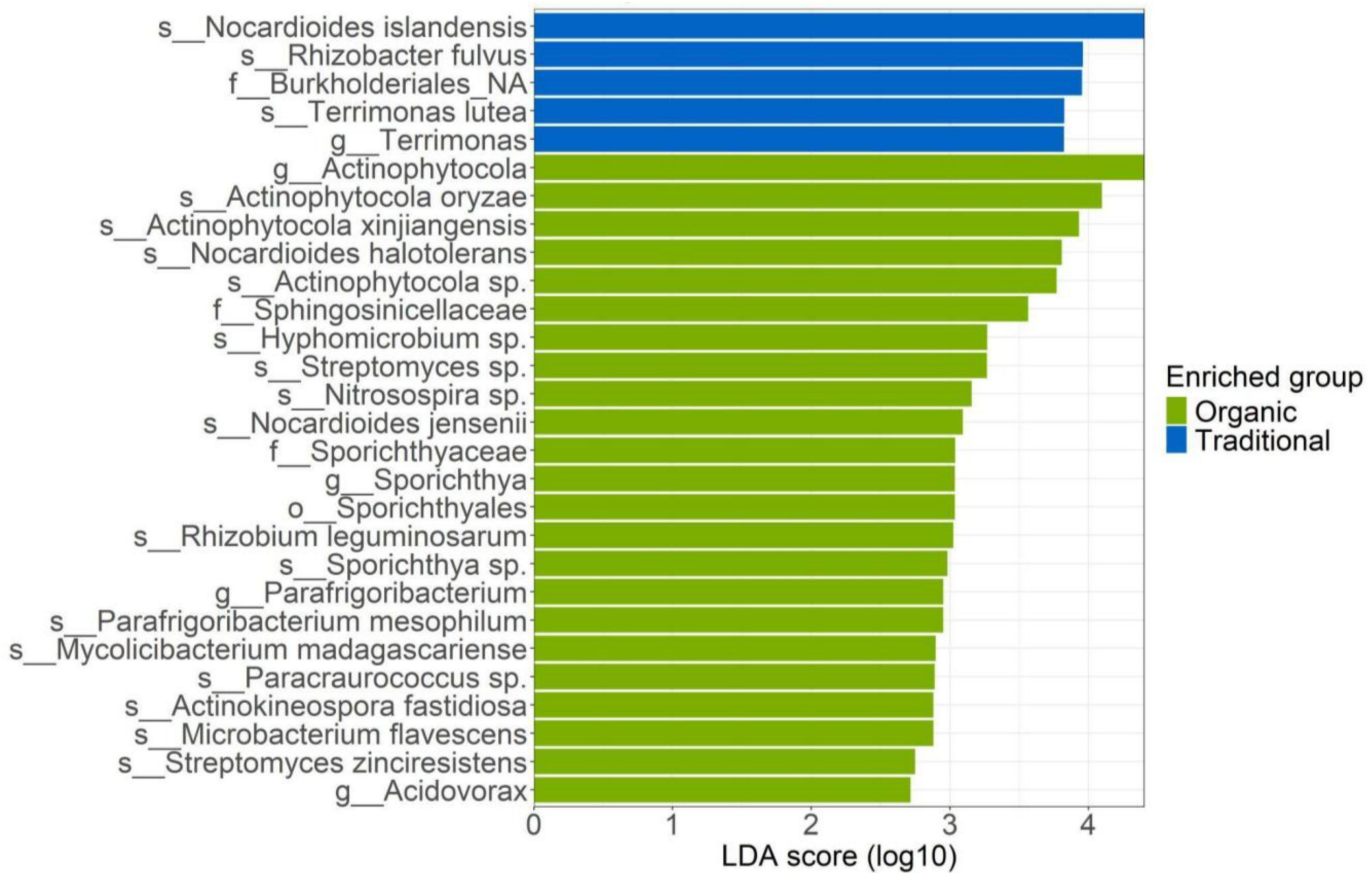
LefSe analysis of the root-associated bacterial community for the three managements: conventional, traditional and organic.

Evaluating the LefSe analysis of the root-associated bacterial community (**Figure 4**), the 28 biomarkers were found to belong exclusively to just two types of management: organic and traditional. Within the traditional management, the differentially abundant biomarkers are represented by the following taxa: *Nocardiodes islandensis* belonging to the phylum *Actinomycetota* with an LDA score of 4.5; the species *Rhizobacter fulvus* and an unknown family belonging to the order *Burkholderioles* with scores of about 4; and finally among the differentially abundant biomarkers in traditional management, the species *Terrimonas lutea* (phylum *Bacteroidota*, genus *Terrimonas*) with an LDA score of more than 3.5 is evident. In the organic management 23 differentially abundant taxa were found by the LefSe analysis, all belonging to the phyla *Actinomycetota* and *Pseudomonadota*. Among the top ten biomarkers reported with the highest score we find genus *Actinophytocola* featured by the species falling within the phylum *Actinomycetota*, such as: *Actinophytocola oryzae*, *Actinophytocola xinjiangensis* and *Actinophytocola* sp. With a score of approximately 4, *Nocardioides halotolerans, Streptomyces* sp. And *Sporichthya jensenii* with a score higher than 3. While among the biomarkers belonging to the phylum *Pseudomonadota*, the family *Sphyngosinicellaceae* with an LDA of 3.5 and the species *Hyphomicrobium* sp. And *Nitrosospira* sp. Were found to have a high score. Other biomarkers with an approximate LDA score of 3 are the species *Sporichtya* sp., *Parafrigoribacterium mesophilum, Actinokineospora fastidiosa, Paracraurococcus* and *Microbacterium favescens* (**Figure 4**).

**Figure 5:**
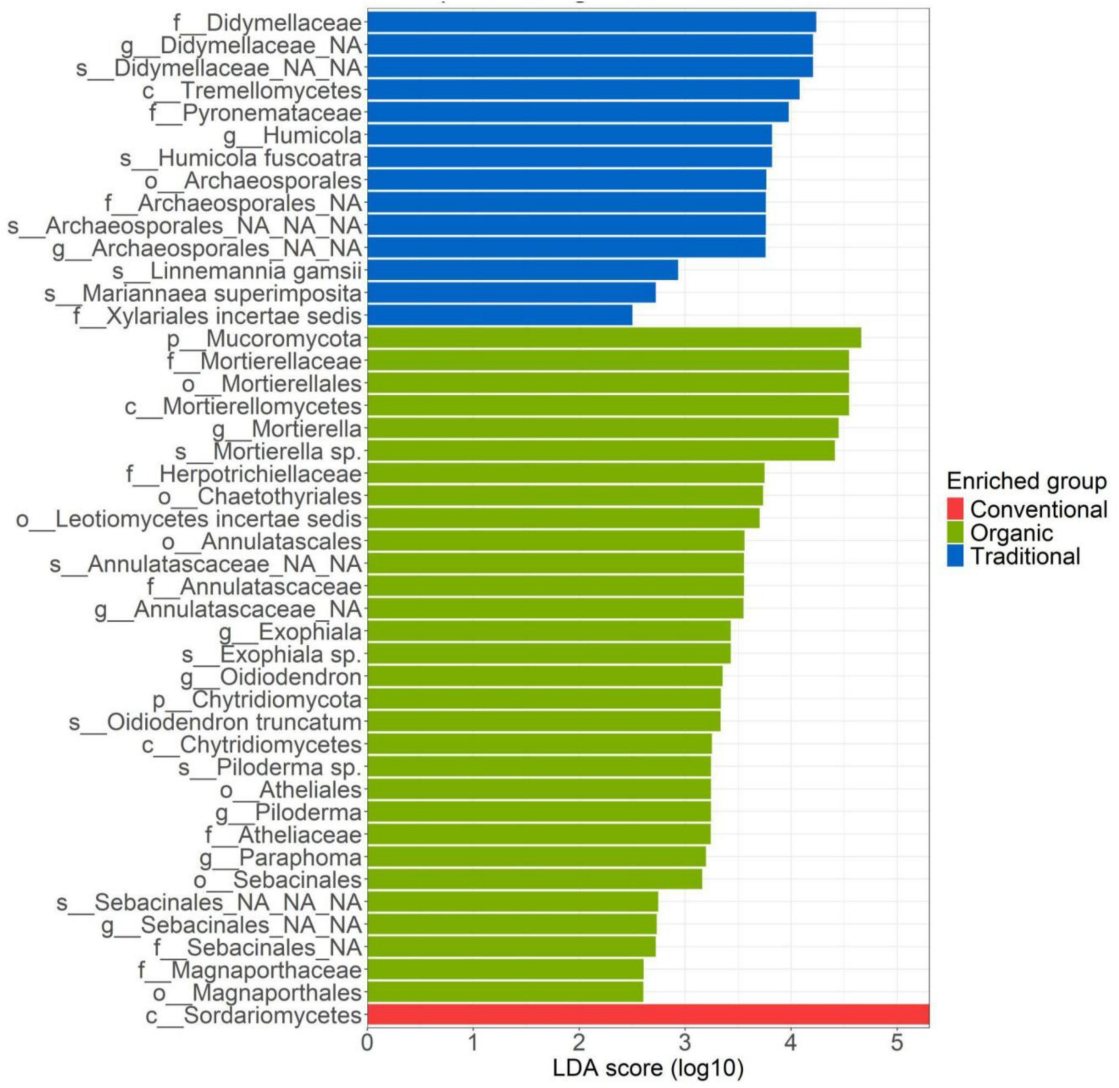
LefSe analysis of the root-associated fungal community for the three managements: conventional, traditional and organic.

The LefSe analysis of the root-associated fungal community (**Figure 5**) identified a total of 45 biomarkers across the three management practices, revealing a clear predominance of taxa from the *Ascomycota* phylum. For the traditional management, the families *Didymellaceae* and *Pyronemataceae* were most prominent, each exhibiting high LDA scores above 4. The genus *Humicola* and the species *Humicola fuscoatra* showed lower LDA scores, below 4. In contrast, the order *Archaeosporales*, along with the species *Linnemania gamsii* and *Mariannaea superimposita*, were differentially abundant, with LDA scores near 3. In conventional management, the LefSe analysis highlighted only a single biomarker: the class *Sardariomycetes* (phylum *Ascomycota*), which had a notably high LDA score greater than 5. For organic management, the majority of biomarkers also belonged to the *Ascomycota* phylum. However, the highest differentially abundant taxa were from the *Mucoromycota* phylum, with an LDA score exceeding 4.5. The next five biomarkers, which were all part of the same taxonomic lineage, exhibited LDA scores around 4.5, and included: *Mortierellamycetes* (class), *Mortierellales* (order), *Mortierellaceae* (family), *Mortierella* (genus), and *Mortierella* sp. In addition, several biomarkers had LDA scores above 3, including the families *Herpotrichiellaceae* and *Annulatascaceae*, the order *Ketothyriales*, and the genera *Exophiala*, *Oidiodendron*, *Paraphoma*, and *Piloderma*. Notably, the species *Exophiala* sp., *Oidiodendron truncatum*, and *Piloderma* sp. Were also found to be differentially abundant. Finally, the orders *Atheliales* and *Sebacinales*, both belonging to the *Basidiomycota* phylum, were also identified as differentially abundant.

### 3.6 Functional classification of the leaf-associated microbiome

Concerning the aboveground bacterial community, the functional classification revealed 34 functions across 64 OTUs (**Supplementary Table S5**).

**Figure 6:**
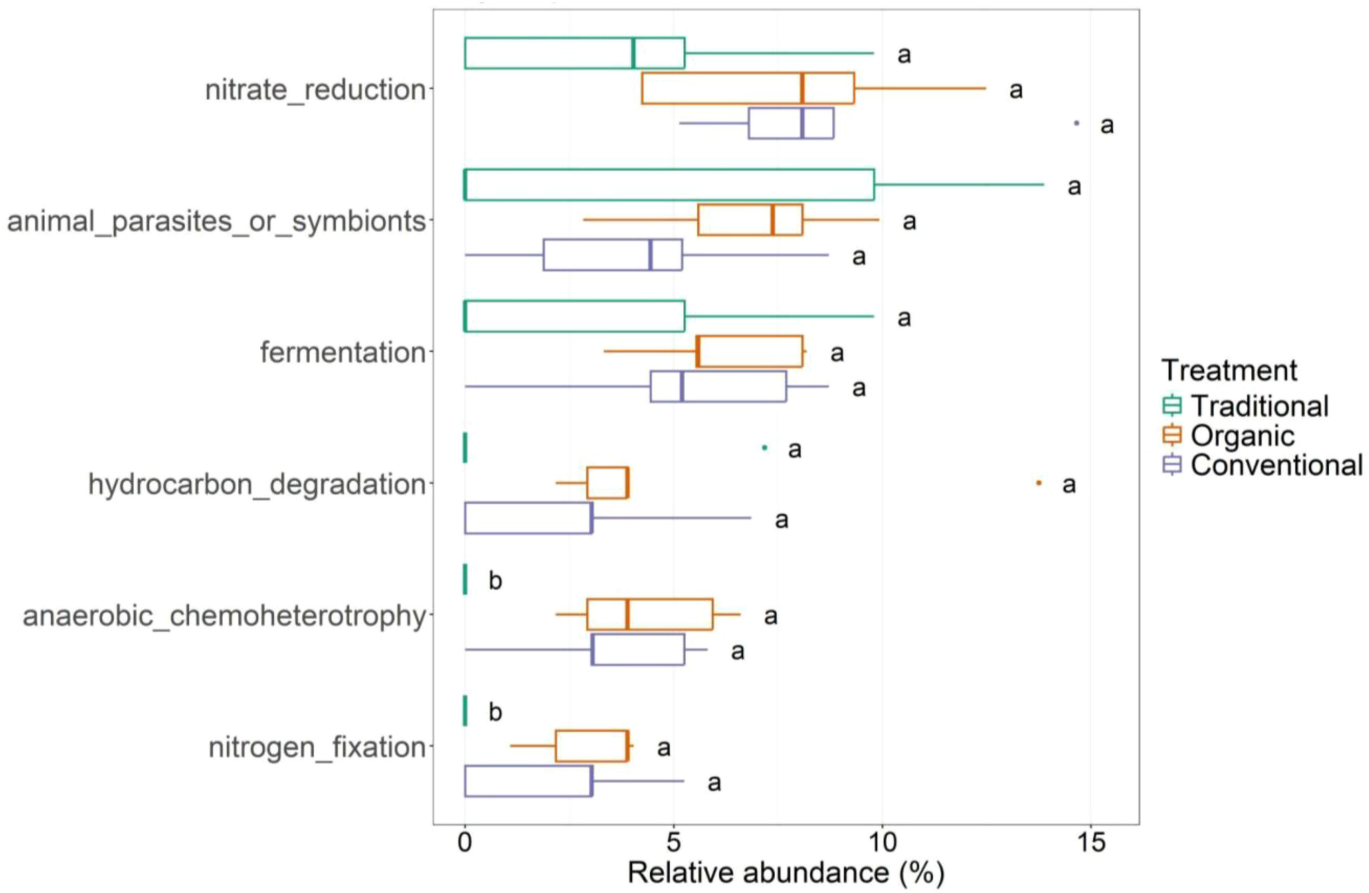
Functional traits of the leaf-associated bacterial community for the three managements: traditional, conventional and organic.

**Figure 6** illustrates that traditional treatments show significant differences in bacterial functions, specifically anaerobic_chemoheterotrophy and nitrogen_fixation, compared to the other management types. Organic management, while only significantly different from traditional management (and not from conventional management), is most strongly associated with both of these functions. The species *Methylocystis heyeri*, identified as a biomarker for organic management in the LefSe analysis (as previously mentioned), is responsible for carrying out both functions. On the other hand, *Rhodococcus erythropolis*, *Enterococcus cecorum* and *Erwinia oleae* are unique species for anaerobic_chemoheterotrophic function, while *Rhizobium leguminosarum* is found to be unique species for nitrogen_fixation function. These species are found to be characteristic of the organic management samples, too. Nitrate_reduction, a function not differentially expressed but relevant in terms of abundance, is realized by *Enhydrobacter aerosaccus* and *Salinibacterium xinjiangense* in both conventional and organic treatments. Functions like animal_parasite_or_symbionts, fermentation, and hydrocarbon_degradation occur more frequently for both conventional and organic methods.

**Figure 7:**
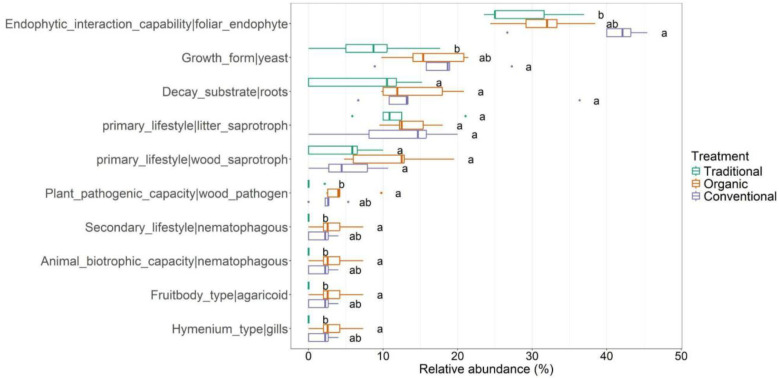
Functional traits of the leaf-associated fungal community for the three managements: traditional, conventional and organic.

The functional traits linked to the leaf-associated fungal community are 78 representative of 123 OTUs (**Supplementary Table S5**). The **Figure 7** indicates that the foliar_endophyte function is the most abundant in the fungal community in conventional treatments and shows differences only with the communities of traditional treatments. Our data reveal that *Alternaria alternata* and *Aureobasidium* sp. Are the taxa to which the above function is associated. The same trend is visible for the growth_form|yeast function carried out mainly from *Bannozyma yamatoana* in conventional treatment, from *Malassezia* sp. In organic treatment and *Vishniacozyma carnescens* in traditional treatment. Functional differences between traditional and organic treatments are found for the following traits: Plant_pathogenic_capacity|wood_pathogen, Secondary_lifestyle|nematophagous, Animal_biotrophic_capacity|nematophagous, Fruitbody_type|agaricoid, Hymenium_type|gills. All these functional traits, due to their unrepresentativeness in traditional treatments, show no significant differences with conventional treatments. The species belonging to the genus *Pleurotus* (*Pleurotus floridanus* and *Pleurotus ostreatus*) perform all these functions. In addition, *Biscogniauxia mediterranea*, *Dendrostoma leiphaemia, Ganoderma adspersum, Vuilleminia comedens*, which are well represented in organic treatments also perform the function of wood_pathogen. Finally, functions Decay_substrate|roots and primary_lifestyle|litter_saprotroph are well represented in terms of relative abundance in the three treatments (particularly in the conventional) although they do not show significant differences. Taxa such as *Cladosporium delicatulum*, particularly abundant in conventional treatments, performs the litter_saprotroph function.

### 3.7 Functional analysis of the root-associated bacterial and fungal community

**Figure 8:**
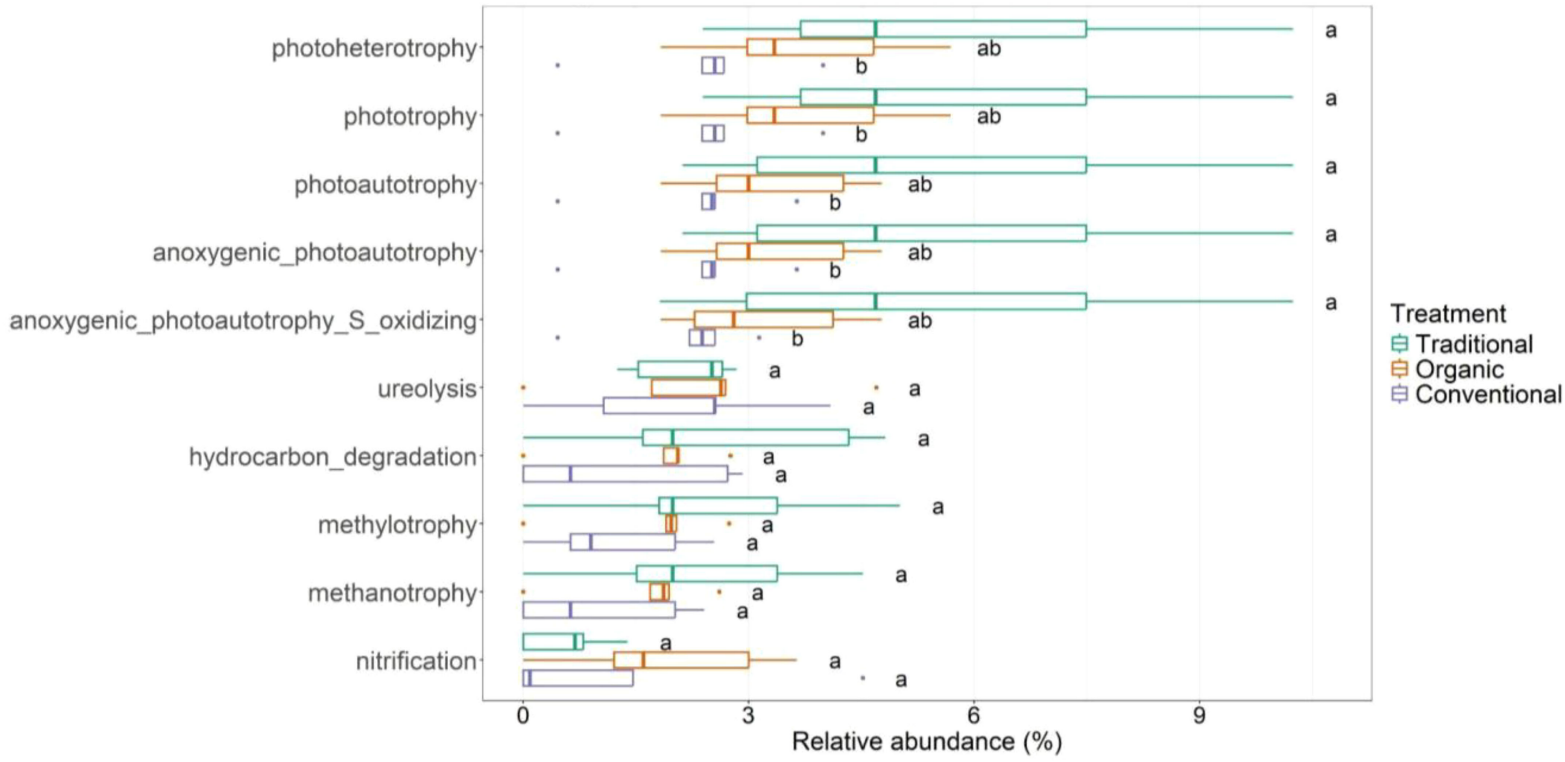
Functional traits of the root-associated bacterial community for the three managements: traditional, conventional and organic; the top 10 functions are shown.

The functional analysis of the root-associated bacterial community (**Figure 8**) reveals 63 distinct functions across 546 OTUs (**Supplementary Table S6**). The first five functional traits are significantly more abundant for traditional management compared to conventional ones. The first two functions (**Figure 8**) are related to photoheterotrophy and phototrophy bacteria, for which *Paracraurococcus* sp. Is responsible. The next functional trait highlighted by FAPROTAX is photoautotrophy, an activity carried out mainly by species such as *Rhodoplanes* sp., *Rhodopseudomonas palustris* and *Rubrivivax gelatinosus*. The same species, *Rhodoplanes* sp. And *Rhodopseudomonas palustris* are implicated in functional traits such as anoxigenic_photoautotrophy and anoxigenic_photoautotrophy_S_oxidising respectively. Other top abundant functions do not show significative differences among management. For instance, ureolysis (with a higher abundance in the organic treatment) is predominantly performed by *Hyphomicrobium* sp. Which is a differentially abundant species of the organic management. Furthermore, another functional trait prevailing in the organic management is nitrification for which the biomarker of biological management *Nitrosospira* sp. Is involved. The other functions highlighted by FAPROTAX are hydrocarbon_degradation, methylotrophy and methanotrophy.

**Figure 9:**
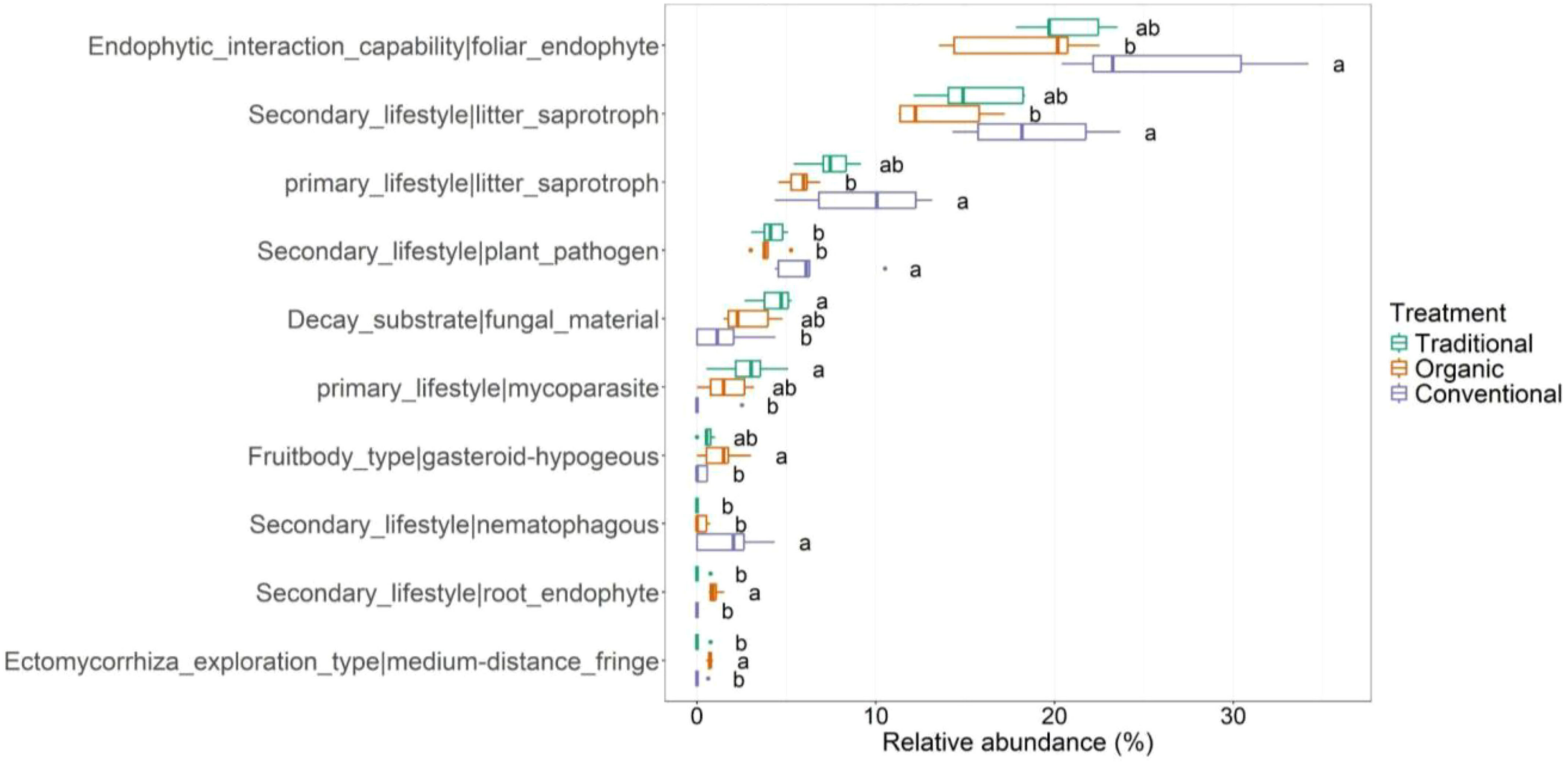
Functional traits of the root-associated fungal community for the three managements: traditional, conventional and organic.

The predictive functional analysis of the root-associated fungal community revealed 191 distinct functions assigned to 473 OTUs (**Supplementary Table S7**). Among the most representative functions (**Figure 9**) we find the function foliar_endophyte in first place and is more abundantly carried out within conventional management. Moreover, it appears to be a significantly more abundant function than organic treatment, while traditional management, although well represented by this function, shows no significant differences with the other two management types. 112 taxa perform this function with 61 of these corresponding to the class *Sordariomycetes*, which is also a biomarker detected by the LefSe analysis in the conventional treatment as shown above. Functional analysis showed that several species of the genus *Trichoderma* (belonging to the class *Sordariomycetes*) perform the foliar_endophyte function. The conventional management shows a higher abundance for the litter_saprotroph function with 140 taxa involved, mostly represented by the *Sordariomycetes* class (with genera such as *Fusarium, Chaetomium* and *Myrothecium*). The conventional management host also a higher abundance of nematophagous fungi, due to the presence *Pleurotus* genus. The representative fungal traits of the traditional management, are Decay_substrate|fungal_materials and Primary_lifestyle|mycoparasite. The class *Tremellomycetes*, which is differentially abundant within the traditional management, appears from the functional analysis to be a taxa involved in both of the above-mentioned functions. Specifically in the mycoparasite function, the genus *Papiliotrema* is linked to perform this function within the class *Tremellomycetes*. With regard to organic management, the function secondary_lifestyle|root_endophyte is significantly higher compared to the other types of management. The species *Oidiodendron tenuissimum* and *Oidiodendrum truncatum* are the main taxa involved in this function for organic groves.

## 4 Discussion

In this study, we investigated to what extent different agricultural management practices could influence belowground and aboveground olive microbiome under different agricultural management. Specifically, organic, conventional and traditional management were investigated with the hypothesis that conventional management may act as an ecological disturbance, potentially influencing both soil and plant health. While numerous studies have explored the olive microbiome (Fernández-González et al., 2019; Kacem, 2023), few of them simultaneously analyze both belowground and aboveground. In addition, we focused on Ortice cultivar (typical of southern Italy) which has not yet been investigated. The data highlighted that community composition shifted in ways that are not captured by richness alone. PERMANOVA revealed significant multivariate differences among treatments, indicating that management affected the overall structure of the communities even when alpha diversity remained largely unchanged. Biomarker analysis further highlighted taxon-specific and biologically relevant responses. Regarding the root-associated microbiome, we observed that the fungal community is significantly influenced by management practices, showing differences between conventional and organic, as well as conventional and traditional (PERMANOVA results). These results are consistent with those of Kacem (2023), who suggests that tillage, a characteristic of conventional management, exerts a greater impact on the fungal community than on the bacterial community in the rhizosphere. The management type also affects aboveground microbiome, for which both fungi and bacterial communities of traditional treatments significantly differ. Concerning of root-associated bacteria, the genus *Actinophytocola* was identified as the top biomarker of organic management. This genus is particularly significant for the fitness of olive trees, contributing to drought tolerance and exhibiting antimicrobial properties that inhibit the growth of well-known human pathogens such as *B. subtilis* and *S. aureus* (Fernández-González et al., 2019). Additionally, the biomarker *Streptomyces* was also present in the organic treatment, recognized as a biocontrol agent (BCA) in various pathosystems (El-Tarabily and Sivasithamparam, 2006; Law et al., 2017). The abundance of *Streptomyces* in the organic management underscores the positive effects of these agronomic practices. In traditional treatment, biomarkers belonging to the *Burkholderiales* order (including the species *Rhizobacter fulvus*) are indicators of organic environments (Liu et al., 2022; Schmidt et al., 2019). Furthermore, another relevant biomarker detected is *Rhizobium leguminosarum*. This species belongs to the *Rhizobiaceae* family reported in literature as a root symbiont useful to the plant for nitrogen fixation activity (Harman and Uphoff, 2019). In contrast, little is yet known about *Nocardioides islandensis* and *Terrimonas* sp. role in the rhizosphere of the olive tree. Concerning the root-associated fungal taxonomy, most of the differentially abundant biomarkers are found to be related to organic management, followed by those of traditional management and finally by conventional management. The class of *Sordariomycetes*, the only biomarker of conventional treatment, includes taxa that perform a wide range of functions. Some are plant pathogens that result in leaf, stem, and root diseases across a wide range of hosts, whereas others cause disease in arthropods and mammals. Many species of *Sordariomycetes* are involved in decomposition and nutrient cycling as saprophytes, in addition to several others that persist in this role (Maharachchikumbura et al., 2016). The biomarkers most abundant in traditional treatment are part of the family *Didymellaceae*. The majority of *Didymellaceae* members were plant pathogens with a wide variety of host species, primarily resulting in damage to the leaves and stems, as stated by Chen et al. (2017). The analysis of aboveground bacteria using LefSe revealed that conventional farming management shows a higher number of biomarkers compared to the other two agricultural practices. Notably, the presence of the *Rhizobiaceae* family stands out. This family includes species that form symbiotic relationships with plants at the root level, allowing them to fix nitrogen, a crucial nutrient for plant growth, while simultaneously deriving nourishment from the plant itself (Harman and Uphoff, 2019; Lindström and Mousavi, 2020). *Rhodocyclaceae* are a common family of endophytic bacteria belonging to the class *Betaproteobacteria* and are found to be dominant in leaf-associated communities. *Rhodocyclaceae* were found to be significantly enriched in the phyllosphere of plants growing in unpolluted soil, which could have an impact on the growth and survival of host plants and on bacteria-bacteria interactions (Li et al., 2020). Among biomarkers of organic management, however, we again find a species belonging to the *Rhizobiaceae* family namely *Rhizobium mesosinicum*. Therefore, it would appear that organic management too, as regarded as conventional one, promotes the growth and development of species in the family *Rhizobiaceae* known for their nitrogen fixation activity (Harman and Uphoff, 2019).

The aboveground fungal communities are characterized by distinct biomarker profiles associated with different soil management practices. Organic management promoted the enrichment of the family *Nectriaceae*, particularly *Fusarium proliferatum* and *Fusarium* sp., suggesting a potential shift toward fungi with both pathogenic and saprophytic roles. *Fusarium* spp., despite being commonly associated with olive tree dieback, can show variable pathogenicity depending on several isolates with weak or no pathogenic potential (Trabelsi et al., 2017). Mondello et al. (2019) research suggests that this fungus may suppress pathogen growth via both antibiosis and direct antagonism, as well as by priming host defense responses. These findings indicate a complex ecological role of *Fusarium*, ranging from pathogenic to potentially beneficial strains. The presence of *Taphrinomycetes*-related taxa further supports the distinct fungal dynamics under organic inputs. In contrast, conventional management favored taxa such as *Alternaria alternata* and *Aureobasidium* sp., commonly associated with phylloplane and stress conditions (Nicoletti et al., 2020). Traditional management was characterized by just one biomarker, namely *Stemphylium solani*., which is known to cause minor foliar diseases in olives (Abdelfattah et al., 2015; Frisullo and Carlucci, 2011), indicating possible weak implications for plant health.

We observed that the conventional system shows enriched leaf-associated bacterial taxa but not root-associated taxa probably related to the above-ground management and pesticide/spray regimes. The conventional management involves the application of foliar sprays (mainly fungicides against the main pathogens of olive plants) which suppress fungal biomass, and prevents fungal biomarker taxa from increasing. When fungal competitors are reduced, new ecological space opens on the leaf surface, allowing certain bacteria to grow more easily. Many of these bacteria are tolerant or opportunistic species that take advantage of the conditions created by the fungicide. Similar enrichment of leaf-associated taxa under conventional management was observed in sugarcane (Khoiri et al., 2021). Concerning the belowground microbiome, the traditional and organic farming fosters a soil environment conducive to a thriving and diverse microbial community, leading to higher detection of both bacterial and fungal biomarkers. Conversely, conventional farming practices suppress microbial biomass and diversity through chemical and physical disturbances, resulting in lower biomarker presence. Similar observations of lower differentiation in conventional rhizosphere compared with organic systems have been reported (Hartmann et al., 2015; Zhang et al., 2025). Overall, the enriched leaf-associated taxa in the conventional system likely reflect aboveground selective pressures, whereas the conventional practices tend to homogenize and weaken microbial communities.

Concerning the functional analyses, organic farming appears to have a stronger positive effect on nutrient recycling processes (e.g., nitrogen cycling) and this result is in line with other research (Wu et al., 2024). We found that the leaf-associated microbiome under traditional management harbors a lower abundance of nitrogen-fixing bacteria compared to both organic and conventional treatments, which likely results from the reduced fertilizer inputs in traditional systems. This limitation may restrict available niches for nitrogen-fixing bacteria (Franche et al., 2009; Gattinger et al., 2012; Vitousek et al., 2002). In contrast, nitrogen fertilization, likely driven by enhanced plant growth and increased nitrogen content in the leaves due to high nitrogen inputs (Darlison et al., 2019), may have promoted the proliferation of specific microbial groups. We also observed a significantly higher capacity for endophytic interactions within the fungal community in both organic and conventional treatments. We observed that the capacity for Endophytic_interaction_capability in the aboveground fungal community is significantly more abundant in conventional treatments. The main involved foliar endophyte taxa are *Alternaria alternata*, *Aureobasidium* sp. And *Cladosporium* sp. For which it is well known the potential pathogenic capacity. This remark involved potential pathogens in conventional treatments which may act as disturbance of natural-biological control of such pathogens. Finally, evaluating the functions in the root-associated compartment, it is interesting to note the presence of the bacterium *Rhodopseudomonas palustris* linked to a higher phototropism trait in traditional managed groves. *R. palustris* is a well-known and long-established biological control agent, and is successfully applied to suppress crop diseases and promote plant growth (Luo et al., 2019). The spontaneous occurrence of this species suggests that non-aggressive agricultural practices can support the adaptation of bacterial communities, which adjust their composition in response to abiotic stress factors. Regarding the endophytic_interaction_capability of root-associated fungi, the class *Sordariomycetes* (the most differentially abundant in the conventional treatment) is mostly involved. In agreement with Marañón-Jiménez et al., 2021, we see that saprotrophic fungi can be a major contributor to the degradation of organic substrates and the application of organic materials to land is a common practice in sustainable agriculture (Repullo et al., 2012). Using FAPROTAX (Louca et al., 2016) provided useful insights into potential functional shifts, but predictions are inherently limited by database coverage, the fraction of unassigned environmental taxa, and the variability of functional traits within taxonomic lineages. Consequently, inferred functions should be interpreted with caution rather than as confirmed activities. Even within these constraints, the analysis clearly highlights functional patterns driven by management practices. Understanding the effects of agro-technical practices on the composition and diversity of soil bacterial and fungal communities remains a complex task, particularly because field studies involve environmental variables that are difficult to fully isolate (e.g., within-orchard variability). Methodological aspects, such as the resolution of sequencing approaches and the use of predicted functional profiles, may also influence the detection and functional interpretation of some microbial taxa. Nonetheless, despite these inherent challenges, our results reveal clear patterns showing how different agricultural management practices (organic, conventional, and traditional) affect both belowground and aboveground microbial communities in the Ortice olive cultivar. These findings provide valuable insights that can inform strategies to support more sustainable and resilient olive cultivation.

## Data Availability

The data presented in the study are deposited in the NCBI depository, accession number PRJNA1286176.

## Supplementary material

Supplementary Figure 1 Rarefaction curves of the microbial communities across belowground samples.

Supplementary Figure 2 Rarefaction curves of the microbial communities across aboveground samples.

Supplementary Figure 3 Relative abundance of Phyla **(A)**, Families **(B)** and Species **(C)** of the aboveground bacterial community in the 15 samples divided by treatment. All taxa with a relative abundance of less than 2% are included in “Other”.

Supplementary Figure 4 Relative abundance of Phyla **(A)**, Families **(B)** and Species **(C)** of the aboveground fungal community in the 15 samples divided by treatment. All taxa with a relative abundance of less than 2% are included in “Other”.

Supplementary Figure 5 Principal Coordinate Analysis (PCoA) plot based on Jaccard’s distance matrix of aboveground bacterial and fungal (**(A, B)** respectively) communities and belowground bacterial and fungal (**(C, D)** respectively) across different treatments (Conventional, Organic, and Traditional).

Supplementary Figure 6 Relative abundance of Phyla **(A)**, Families **(B)** and Species **(C)** of the belowground bacterial community in the 15 samples divided by treatment. All taxa with a relative abundance of less than 2% are included in “Other”.

Supplementary Figure 7 Relative abundance of Phyla **(A)**, Families **(B)** and Species **(C)** of the belowground fungal community in the 15 samples divided by treatment. All taxa with a relative abundance of less than 2% are included in “Other”.

Supplementary Table 1 Physico-chemical properties of the analysed soils under different management practices. Values are presented as mean. ANOVA results are included, with F-statistics and corresponding p-values indicating significant differences among the three management types.

Supplementary Table 2 Complete taxonomy and abundance of bacterial and fungal OTUs of the leaf-associated microbiome for each sample.

Supplementary Table 3 Average distances to centroid and permutation test p-values for bacterial and fungal within management (Conventional, Organic, Traditional). Values represent the mean distance of samples to their group centroid, with associated p-values from *betadisper* (*vegan* package) permutation tests assessing differences in dispersion within treatments.

Supplementary Table 4 Complete taxonomy and abundance of bacterial and fungal OTUs of the root-associated microbiome for each sample.

Supplementary Table 5 Functional classification of leaf-associated bacterial OTUs.

Supplementary Table 6 Functional classification of leaf-associated fungal OTUs.

Supplementary Table 7 Functional classification of root-associated bacterial OTUs.

Supplementary Table 8 Functional classification of root-associated fungal OTUs.
