# Supplementary material for: Management affects the diversity and functions of root and leaf-associated microbiomes: implications for olive resilience
Source: Front Plant Sci. 2026 Jan 20;16:1699667. doi: 10.3389/fpls.2025.1699667 (PMC12864071; doi:10.3389/fpls.2025.1699667)

**Supplementary Material**

Figure S1 – Rarefaction curves of the microbial communities across belowground samples


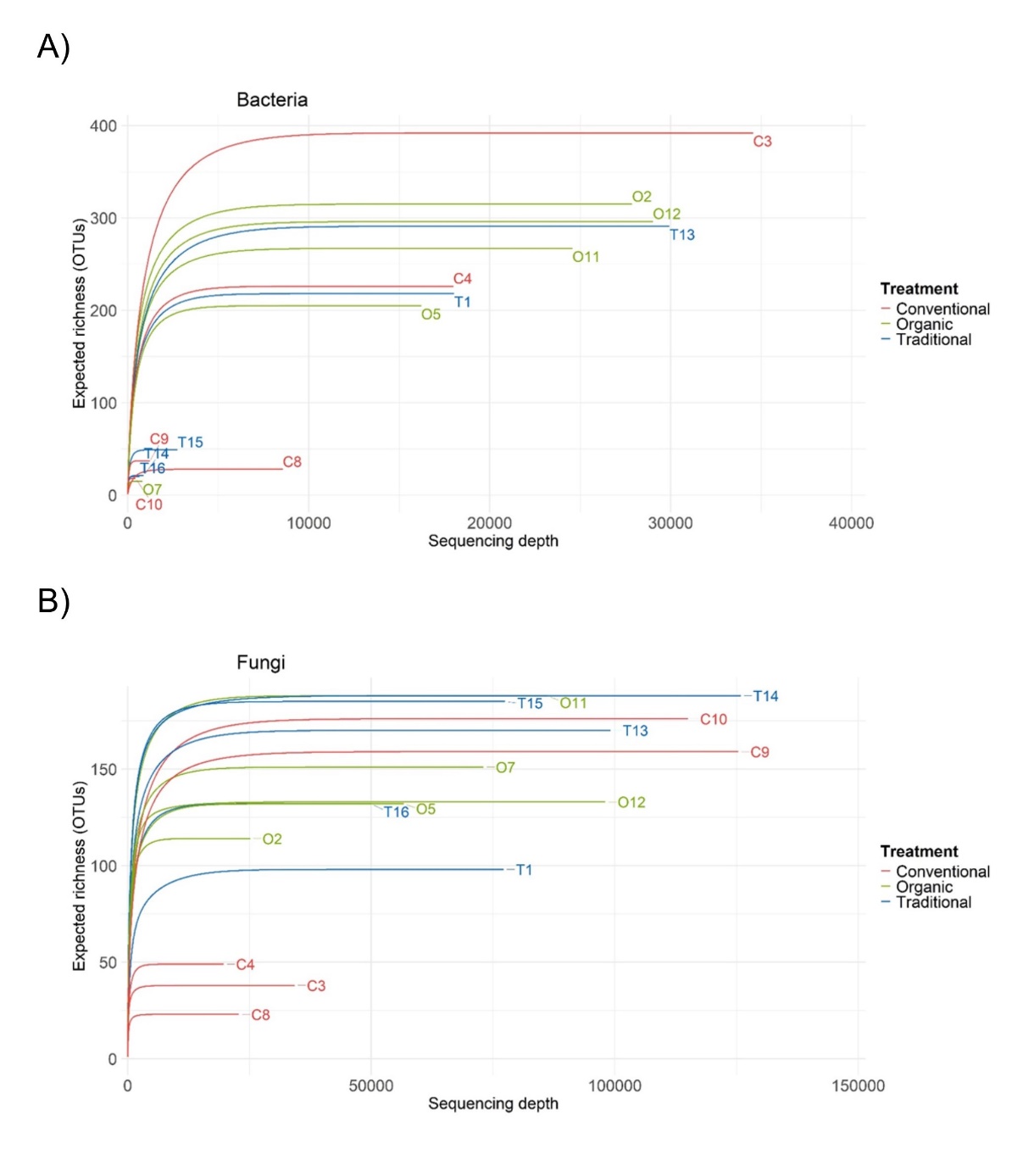


Figure S2 – Rarefaction curves of the microbial communities across aboveground samples


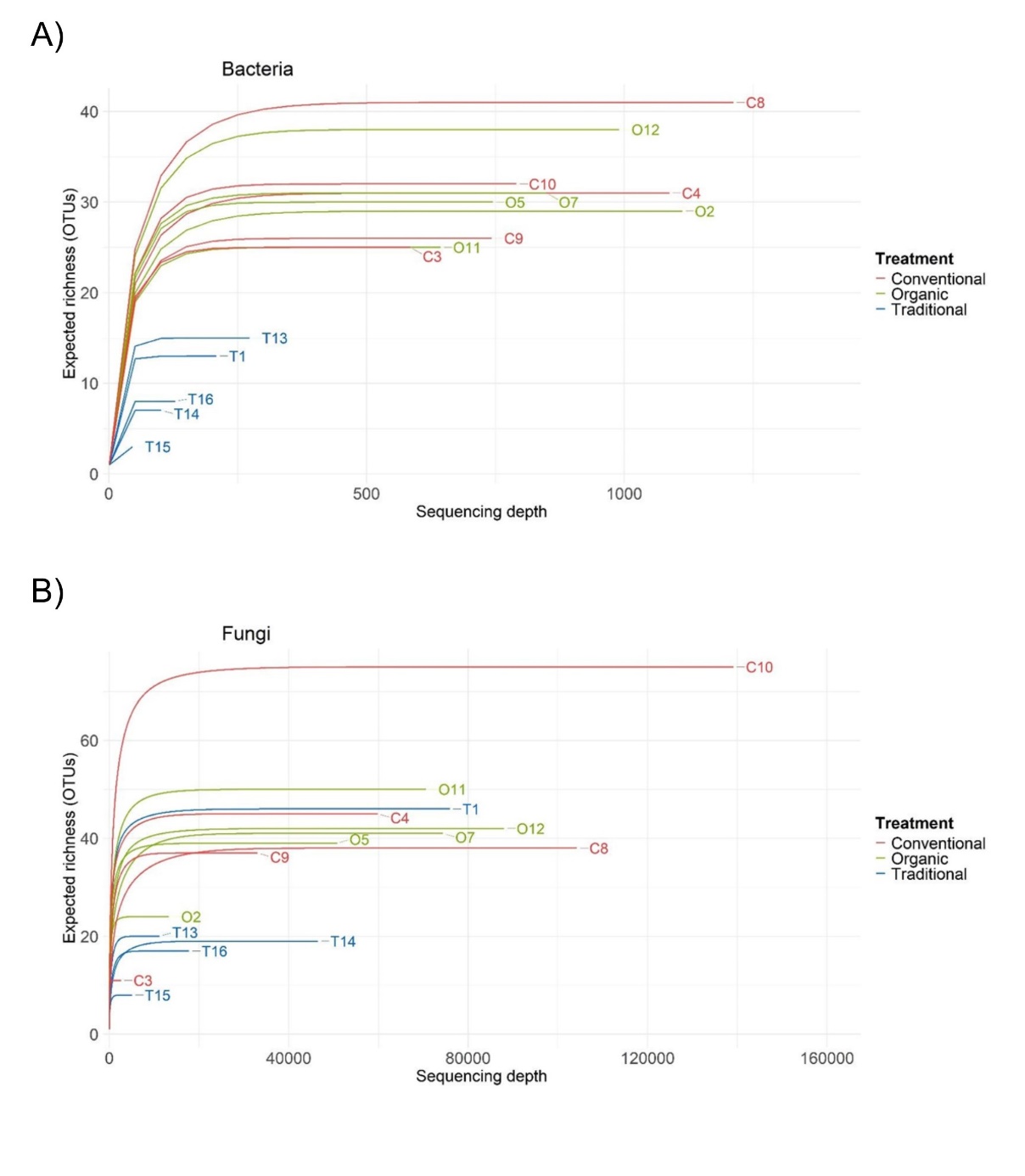


Figure S3 – Relative abundance of Phyla (A), Families (B) and Species (C) of the aboveground bacterial community in the 15 samples divided by treatment. All taxa with a relative abundance of less than 2% are included in “Other”.


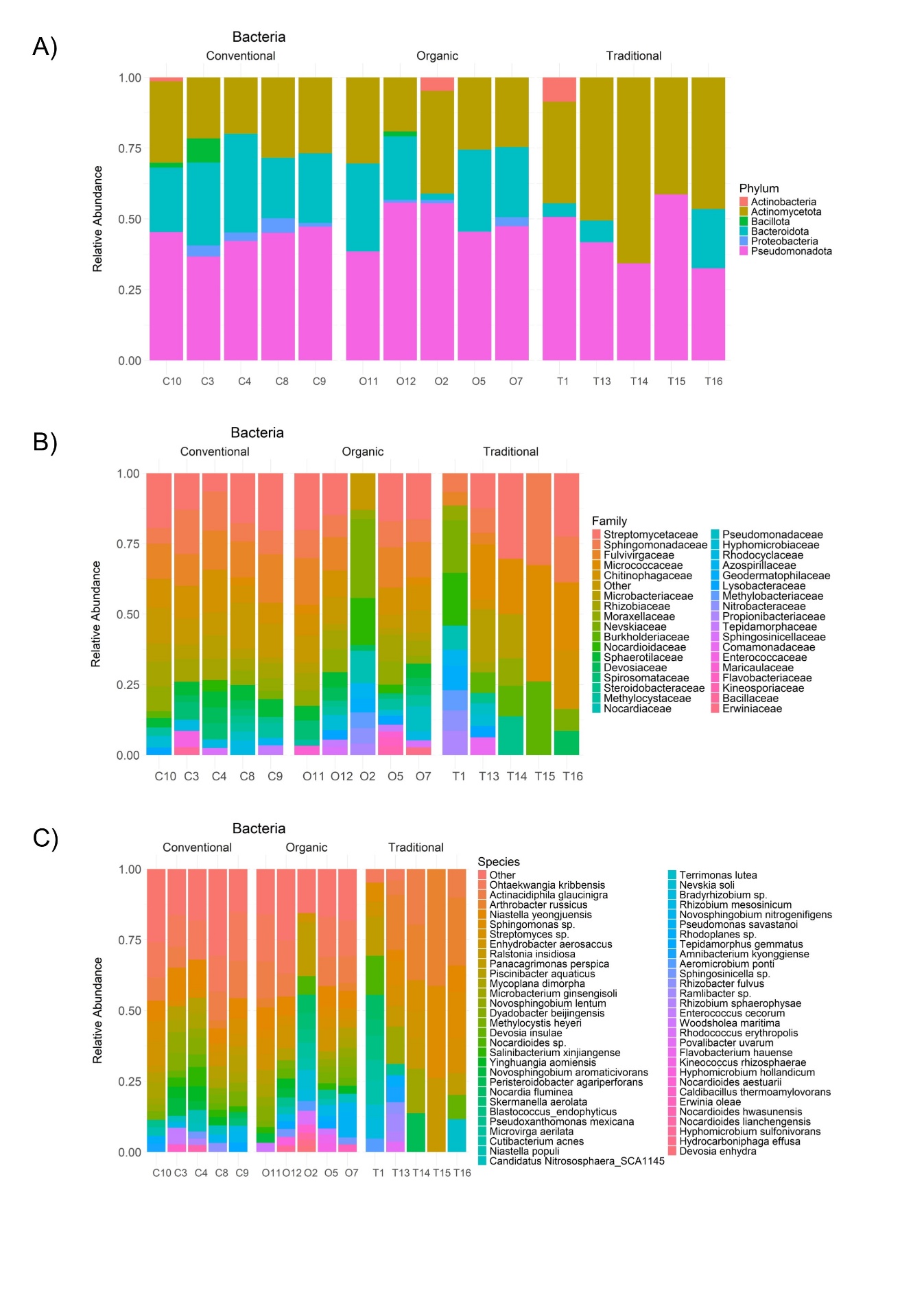


Figure S4 – Relative abundance of Phyla (A), Families (B) and Species (C) of the aboveground fungal community in the 15 samples divided by treatment. All taxa with a relative abundance of less than 2 % are included in “Other”.


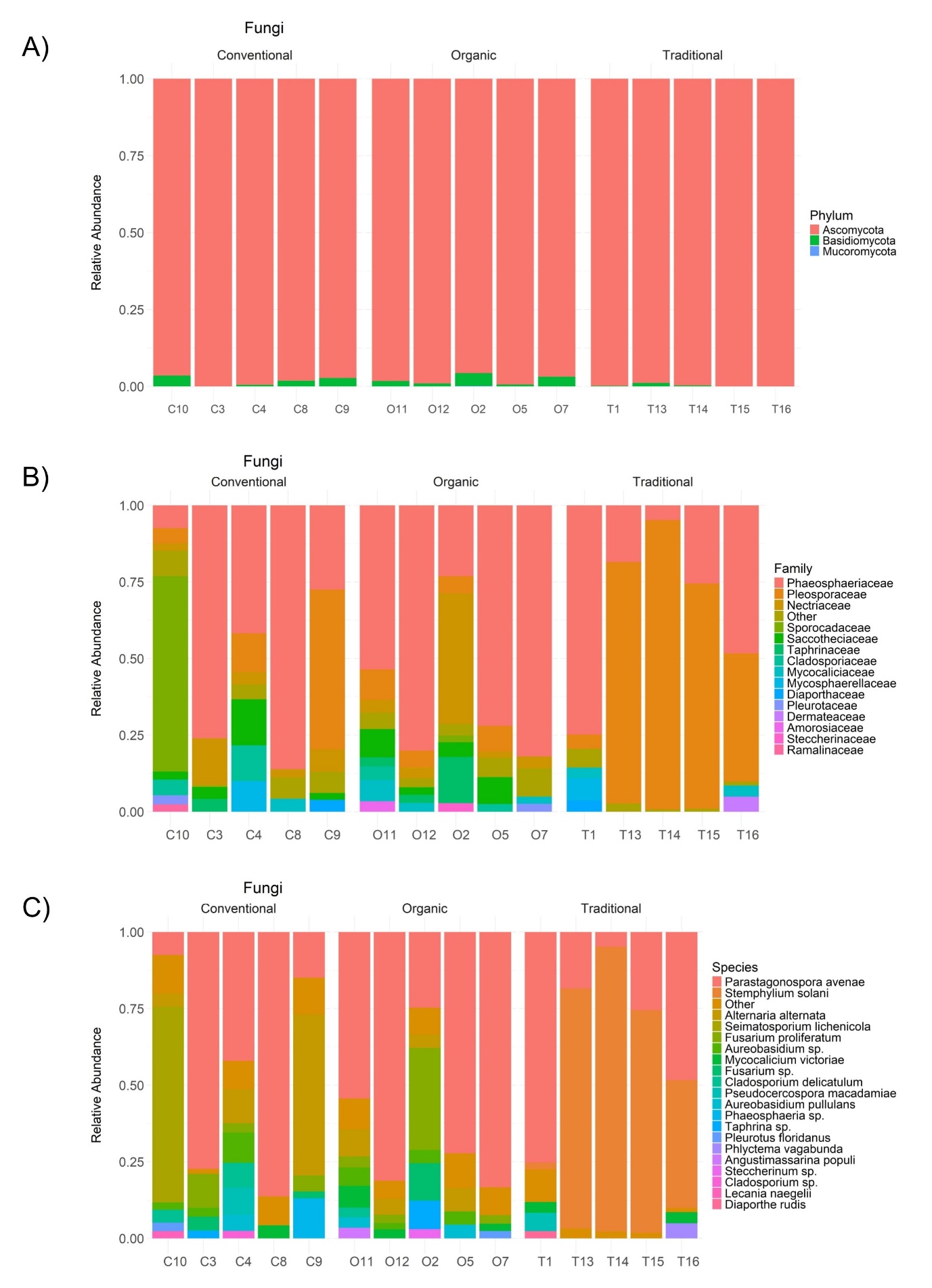


Figure S5 - Principal Coordinate Analysis (PCoA) plot based on Jaccard's distance matrix of aboveground bacterial and fungal (A and B, respectively) communities and belowground bacterial and fungal (C and D, respectively) across different treatments (Conventional, Organic, and Traditional)


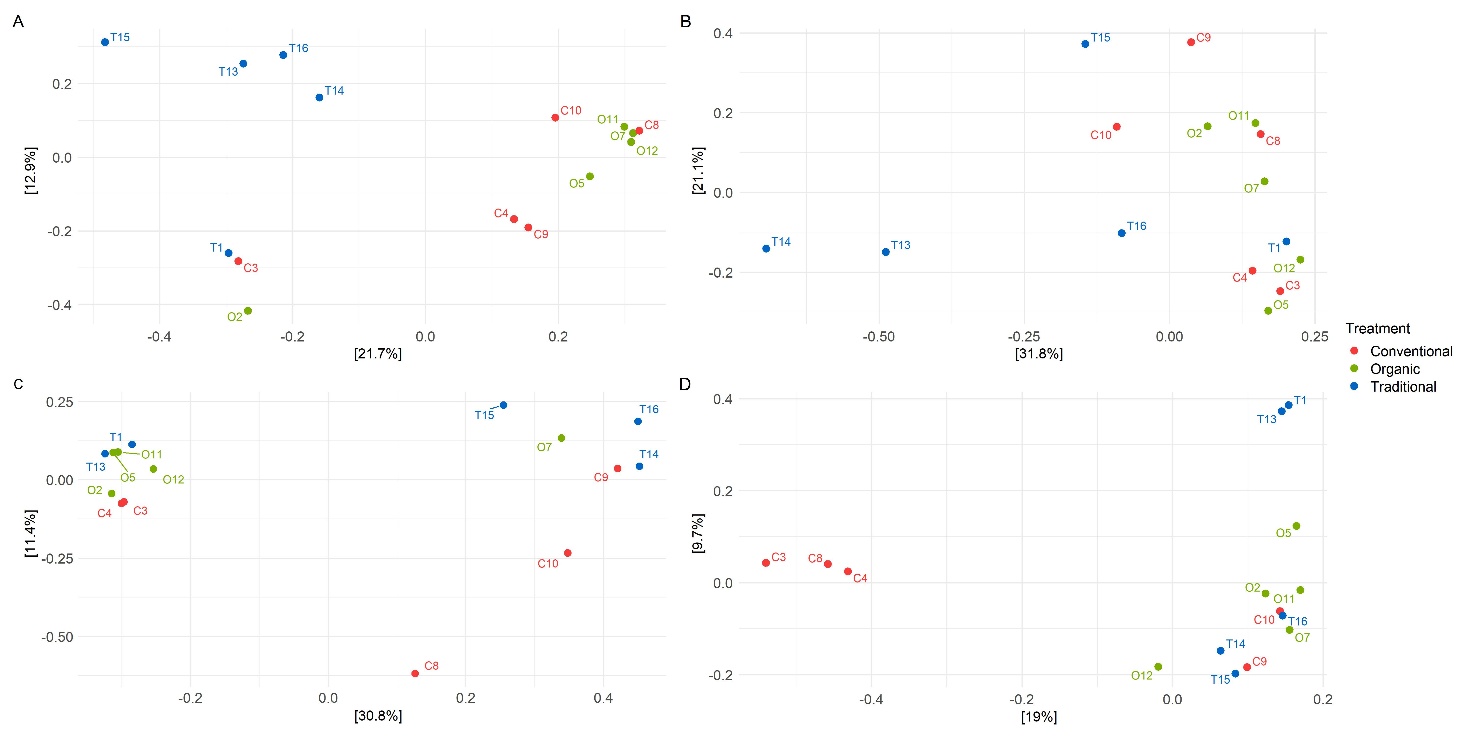


Figure S6 – Relative abundance of Phyla (A), Families (B) and Species (C) of the belowground bacterial community in the 15 samples divided by treatment. All taxa with a relative abundance of less than 2 % are included in “Other”.


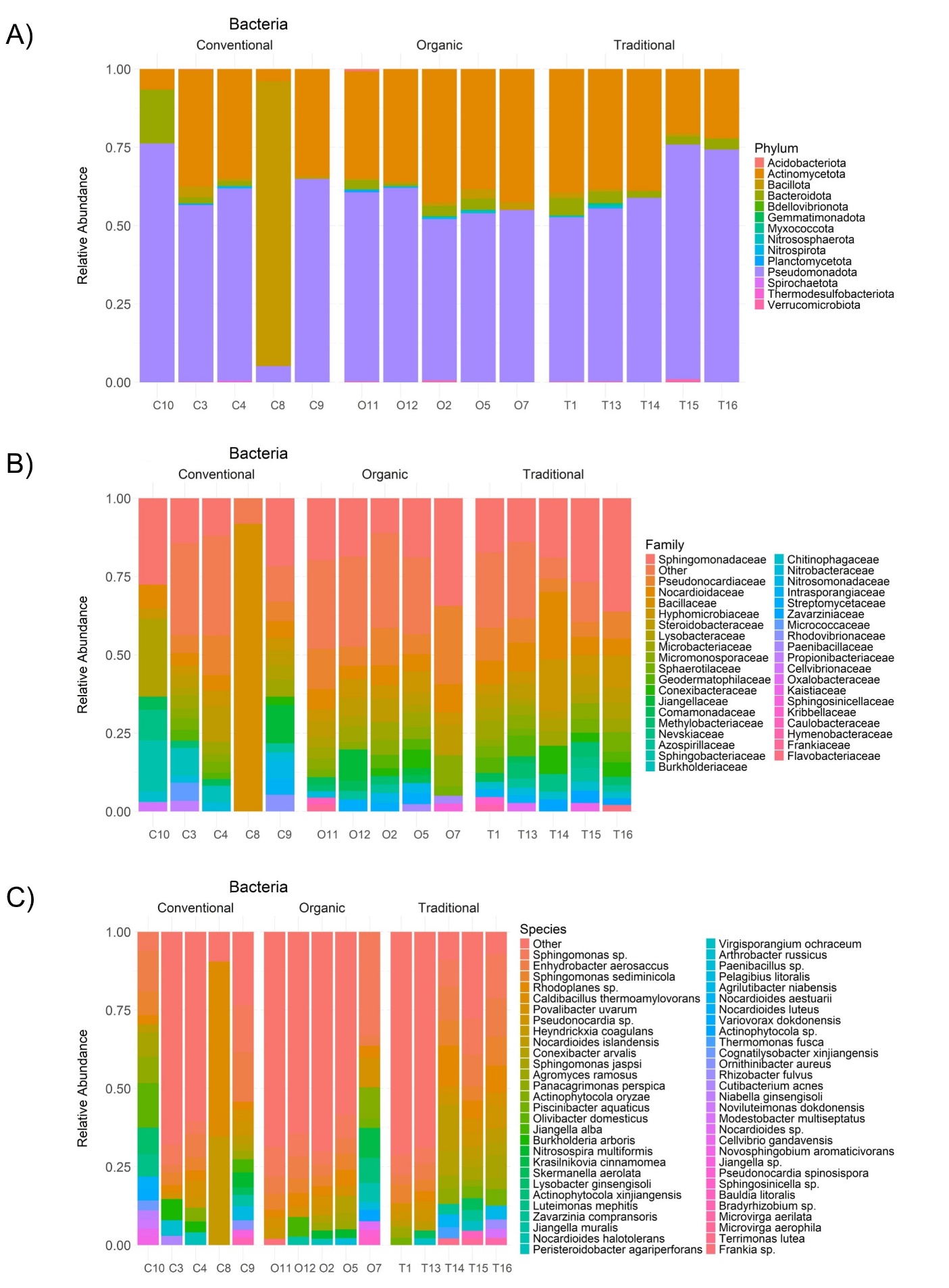


Figure S7 - Relative abundance of Phyla (A), Families (B) and Species (C) of the belowgorund fungal community in the 15 samples divided by treatment. All taxa with a relative abundance of less than 2 % are included in “Other”.


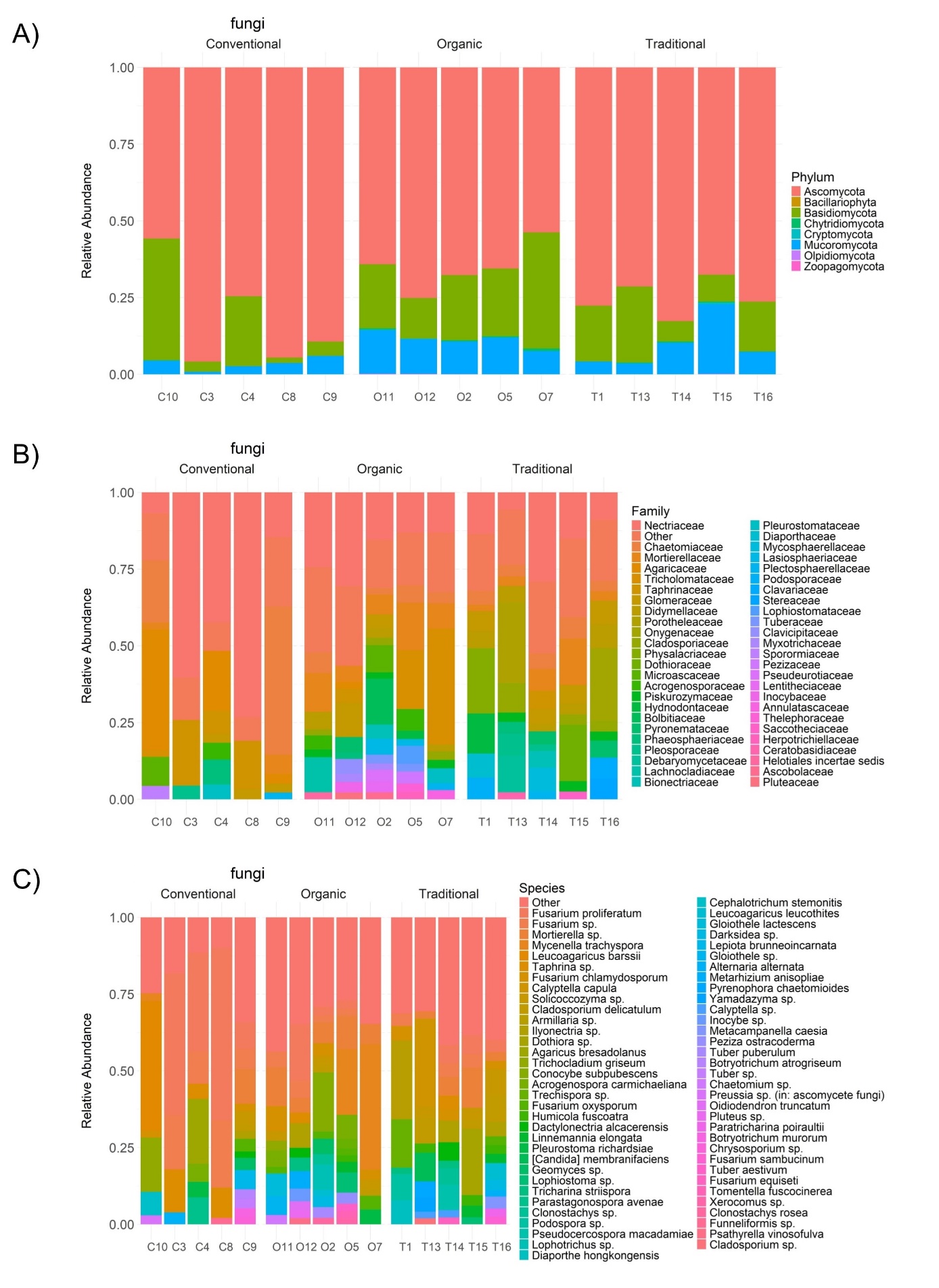

Supplement: Supplementary Figure 1 — Rarefaction curves of the microbial communities across belowground samples. [file SupplementaryFile1.zip › Supplementary Material/Table 1.DOCX]
