# Supplementary material for: Management affects the diversity and functions of root and leaf-associated microbiomes: implications for olive resilience
Source: Front Plant Sci. 2026 Jan 20;16:1699667. doi: 10.3389/fpls.2025.1699667 (PMC12864071; doi:10.3389/fpls.2025.1699667)
Supplement: Supplementary Figure 1 — Rarefaction curves of the microbial communities across belowground samples. [file SupplementaryFile1.zip › Supplementary Material/Table 4.DOCX]

Table S2 - Average distances to centroid and permutation test p-values for bacterial and fungal within management (Conventional, Organic, Traditional). Values represent the mean distance of samples to their group centroid, with associated p-values from *betadisper* (*vegan* package) permutation tests assessing differences in dispersion within treatments.

|  | | **Treatment** | **Mean distance to centroid** |
| --- | --- | --- | --- |
| **Leaf-associated** | **Bacteria** | Conventional | 0,31 |
|  |  | Organic | 0,35 |
|  |  | Traditional | 0,46 |
|  |  | p value | 0,44 |
|  | **Fungi** | Conventional | 0,46 |
|  |  | Organic | 0,30 |
|  |  | Traditional | 0,45 |
|  |  | p value | 0,37 |
| **Root-associated** | **Bacteria** | Conventional | 0,57 |
|  |  | Organic | 0,39 |
|  |  | Traditional | 0,48 |
|  |  | p value | 0,24 |
|  | **Fungi** | Conventional | 0,47 |
|  |  | Organic | 0,43 |
|  |  | Traditional | 0,41 |
|  |  | p value | 0,72 |

Table S2: Values of the average distance from the centroid and p-value of permutation test for leaf and root associated bacterial and fungal communities.
